# Tregs integrate native and CAR-mediated costimulatory signals for control of allograft rejection

**DOI:** 10.1172/jci.insight.167215

**Published:** 2023-10-09

**Authors:** Isaac Rosado-Sánchez, Manjurul Haque, Kevin Salim, Madeleine Speck, Vivian C.W. Fung, Dominic A. Boardman, Majid Mojibian, Giorgio Raimondi, Megan K. Levings

**Affiliations:** 1 BC Children’s Hospital Research Institute, Vancouver, British Columbia, Canada.; 2 School of Biomedical Engineering and; 3 Department of Surgery, University of British Columbia, Vancouver, British Columbia, Canada.; 4 Department of Plastic and Reconstructive Surgery, Johns Hopkins University School of Medicine, Baltimore, Maryland, USA.

**Keywords:** Immunology, Transplantation, Costimulation, Immunotherapy, Tolerance

## Abstract

Tregs expressing chimeric antigen receptors (CAR-Tregs) are a promising tool to promote transplant tolerance. The relationship between CAR structure and Treg function was studied in xenogeneic, immunodeficient mice, revealing advantages of CD28-encoding CARs. However, these models could underrepresent interactions between CAR-Tregs, antigen-presenting cells (APCs), and donor-specific Abs. We generated Tregs expressing HLA-A2–specific CARs with different costimulatory domains and compared their function in vitro and in vivo using an immunocompetent model of transplantation. In vitro, the CD28-encoding CAR had superior antigen-specific suppression, proliferation, and cytokine production. In contrast, in vivo, Tregs expressing CARs encoding CD28, ICOS, programmed cell death 1, and GITR, but not 4-1BB or OX40, all extended skin allograft survival. To reconcile in vitro and in vivo data, we analyzed effects of a CAR encoding CD3ζ but no costimulatory domain. These data revealed that exogenous costimulation from APCs can compensate for the lack of a CAR-encoded CD28 domain. Thus, Tregs expressing a CAR with or without CD28 are functionally equivalent in vivo, mediating similar extension of skin allograft survival and controlling the generation of anti–HLA-A2 alloantibodies. This study reveals a dimension of CAR-Treg biology and has important implications for the design of CARs for clinical use in Tregs.

## Graphical abstract

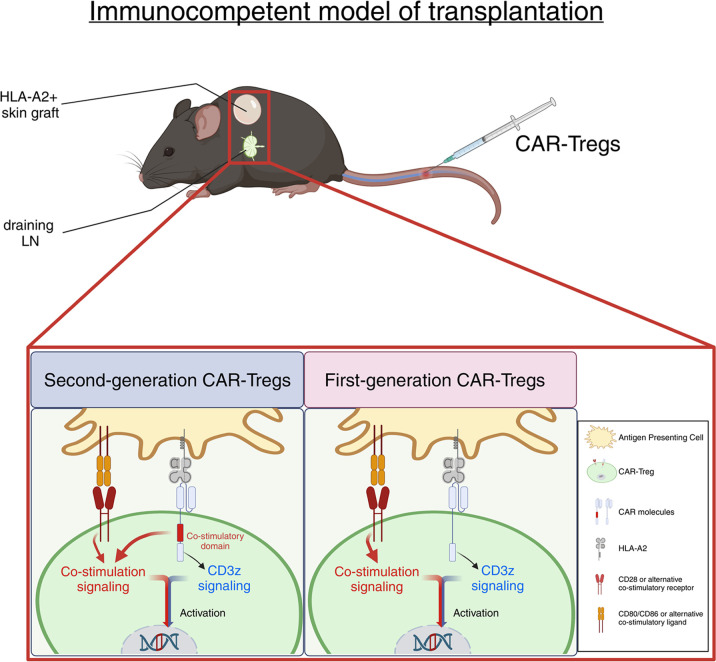

## Introduction

Adoptive cell therapy using Tregs has emerged as a promising therapeutic strategy to promote transplant tolerance and reduce dependence on immunosuppression (1–3). Multiple clinical studies have demonstrated that polyclonal Treg therapy is feasible, safe, and possibly effective (4–7). However, data from preclinical models revealed that alloantigen-specific Tregs are significantly more potent at reducing transplant rejection (8, 9). We and others developed a strategy to generate antigen-specific Tregs using chimeric antigen receptors (CARs), synthetic fusion proteins that redirect T cell specificity. CAR-Tregs are more effective than polyclonal Tregs at limiting xenogeneic graft-versus-host disease (xenoGVHD) (10–12), as well as skin and heart transplant rejection (13–17), and have rapidly transitioned to clinical testing with 2 ongoing phase I/IIa clinical trials (ClinicalTrials.gov NCT04817774 and NCT05234190) (18).

CARs typically comprise an extracellular single-chain Ab (scFv) domain, a hinge, a transmembrane (TM) domain, and customizable intracellular signaling domains. They have been extensively studied in the context of cancer immunotherapy, initially as so-called first-generation CARs encoding only a CD3ζ domain, and subsequently as second- or third-generation CARs adding 1 or more costimulatory domains, respectively (19, 20). In the context of transplantation, the optimal CAR design for Tregs is still under debate (8, 21). We recently explored the function of CARs encoding different costimulatory domains in human Tregs using an immunodeficient mouse model of xenoGVHD and demonstrated that a second-generation CD28-CD3ζ–encoding CAR was optimal in terms of Treg potency, stability, and persistence (10). Similar results were found in other studies using PBMC-reconstitution–based, humanized mouse and skin xenograft models (11, 16). However, drawing clinically relevant conclusions is complicated in these models because of their immunodeficient state and because PBMC reconstitution primarily results in T cell engraftment, with poor or no reconstitution of antigen-presenting cells (APCs), including B cells and DCs (22–25).

Suppressing the ability of APCs to activate effector T cells is a primary mechanism by which Tregs maintain peripheral tolerance (2, 26). Tregs suppress APCs using a range of strategies, including cytotoxic T lymphocyte–associated protein 4–mediated (CTLA-4–mediated) transendocytosis of CD80/86 (27, 28), trogocytosis of MHC class II (29, 30), suppression of cytokine production (31), and induction of death (32–34). Tregs also control the generation of donor-specific anti-HLA Abs (DSAs) by directly suppressing B cell function (35, 36), inducing B cell apoptosis (35), and/or inhibiting follicular helper cells (37–41). APCs (42–44) and DSAs (45, 46) both have critical roles in transplant rejection, so identifying the optimal CAR-Treg design to regulate these cells and processes is an important outstanding question (47).

In this study, we used a mouse model of HLA-A2^+^ skin transplantation to study the structure–function relationship of CAR-Tregs. HLA-A2–specific CARs with different costimulatory domains were expressed in Tregs and studied in vitro and in vivo in an immunocompetent setting. We explored how CAR-Tregs integrate signals from exogenous and endogenous sources and how signal origin shapes function.

## Results

### Generation of costimulation domain variant CAR-Tregs in mice.

**Figure 1:**
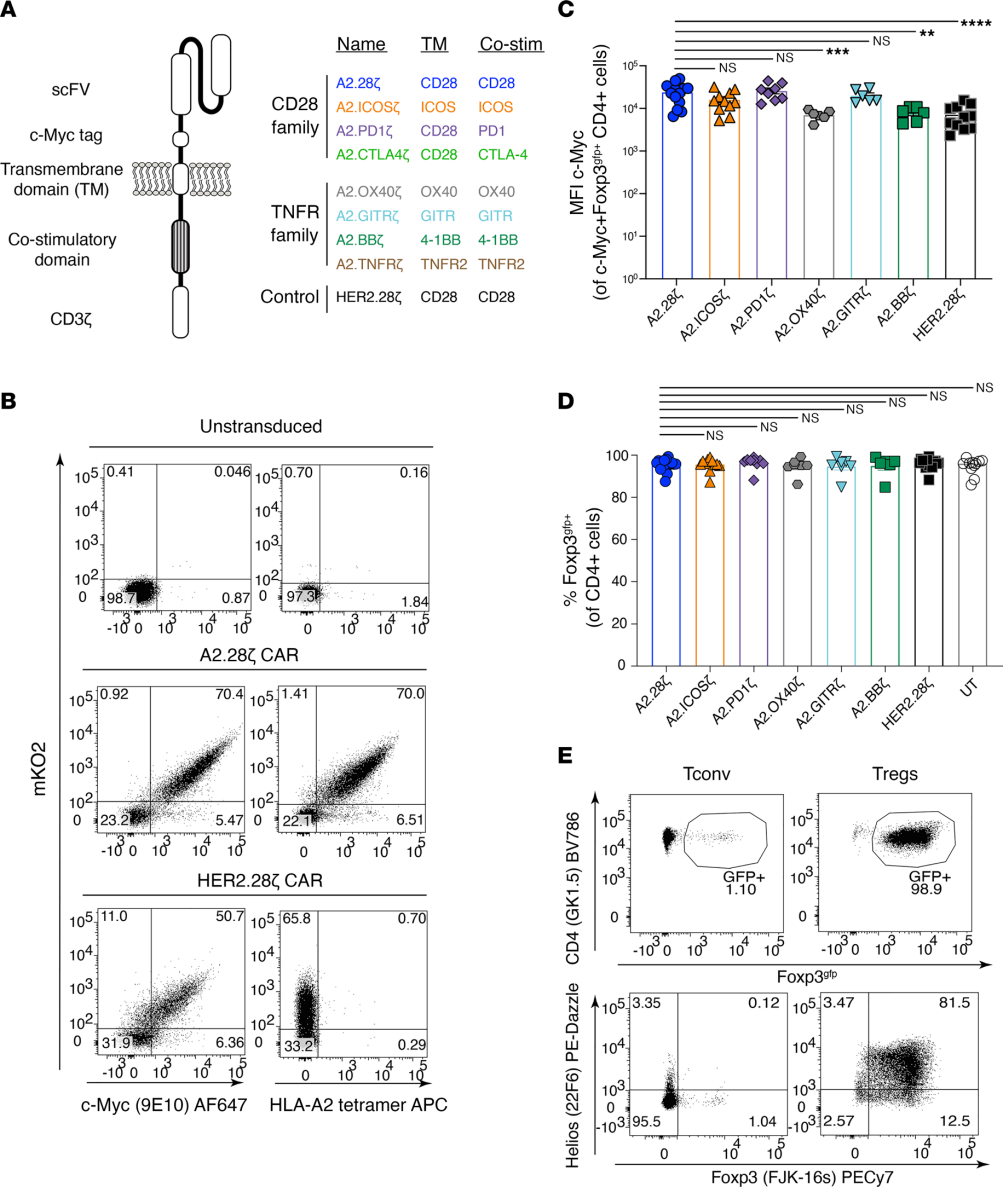
Design and expression of costimulatory domain CAR variants. (**A**) Schematic diagram summarizing the TM and signaling domains incorporated into the second-generation CAR variants. (**B**) Representative flow cytometry plots of at least 3 independent experiments showing CAR (c-Myc) and mKO2 reporter expression and binding to an HLA-A2 tetramer, with percentages shown in corners. (**C**) MFI of CAR expression for different CAR variants in Tregs after expansion gated on live c-Myc^+^CD4^+^Foxp3^gfp+^ cells; *n* = 6–13 replicates from at least 8 independent experiments. (**D**) Foxp3^gfp^ expression in Tregs after expansion, gated on live CD4^+^ cells; *n* = 6–16 replicates from at least 11 independent experiments. (**E**) Representative data of at least 5 independent experiments showing intracellular Foxp3 and Helios expression in CAR-Tregs and control conventional T cells (Tconv) after expansion, gated on total live CD4^+^ cells. Data reported as mean ± SEM. Statistical significance was determined using 1-way ANOVA with a Holm-Šidak posttest. **P < 0.01, ***P < 0.001, ****P < 0.0001. Co-stim, costimulatory; UT, untransduced.

We generated 8 HLA-A2–specific CAR variants containing different TM and costimulatory domains derived from CD28 and TNFR family proteins that have relevance to Treg biology (48) (Figure 1A). Guided by previous studies, TM domains were derived either from CD28 or the intracellular costimulatory protein under investigation (10). CAR variants were cloned into a bicistronic retroviral vector upstream of a monomeric Kusabira-Orange 2 (mKO2) reporter. CD4^+^CD8^–^Thy1.1^+^Foxp3^gfp+^ Tregs were sorted, polyclonally stimulated, transduced, and expanded (Supplemental Figure 1; supplemental material available online with this article; https://doi.org/10.1172/jci.insight.167215DS1). Control Tregs and conventional T cells were expanded in a similar manner but transduced with an irrelevant CAR or left untransduced.

CAR expression and Treg transduction were measured by expression of the CAR-encoded extracellular c-Myc tag and mKO2, respectively (Figure 1B and Supplemental Figure 2A). With the exception of CTLA-4– and TNFR2-encoding CARs (Supplemental Figure 2B, not analyzed further), CAR variants were detected on the cell surface and bound to HLA-A2 tetramers (Figure 1B). After expansion, on average, approximately 70% of cells expressed a CAR (Supplemental Figure 2C). Expression levels of OX40- and 4-1BB–encoding CARs, and the control HER2 CAR, were lower than that of the CD28-encoding CAR (Figure 1C), but there were no differences in gfp (Foxp3^gpf^) or intracellular Foxp3 expression, demonstrating high Treg purity following transduction and expansion (Figure 1, D and E).

### Costimulatory CAR variants differ in their ability to stimulate Tregs.

**Figure 2:**
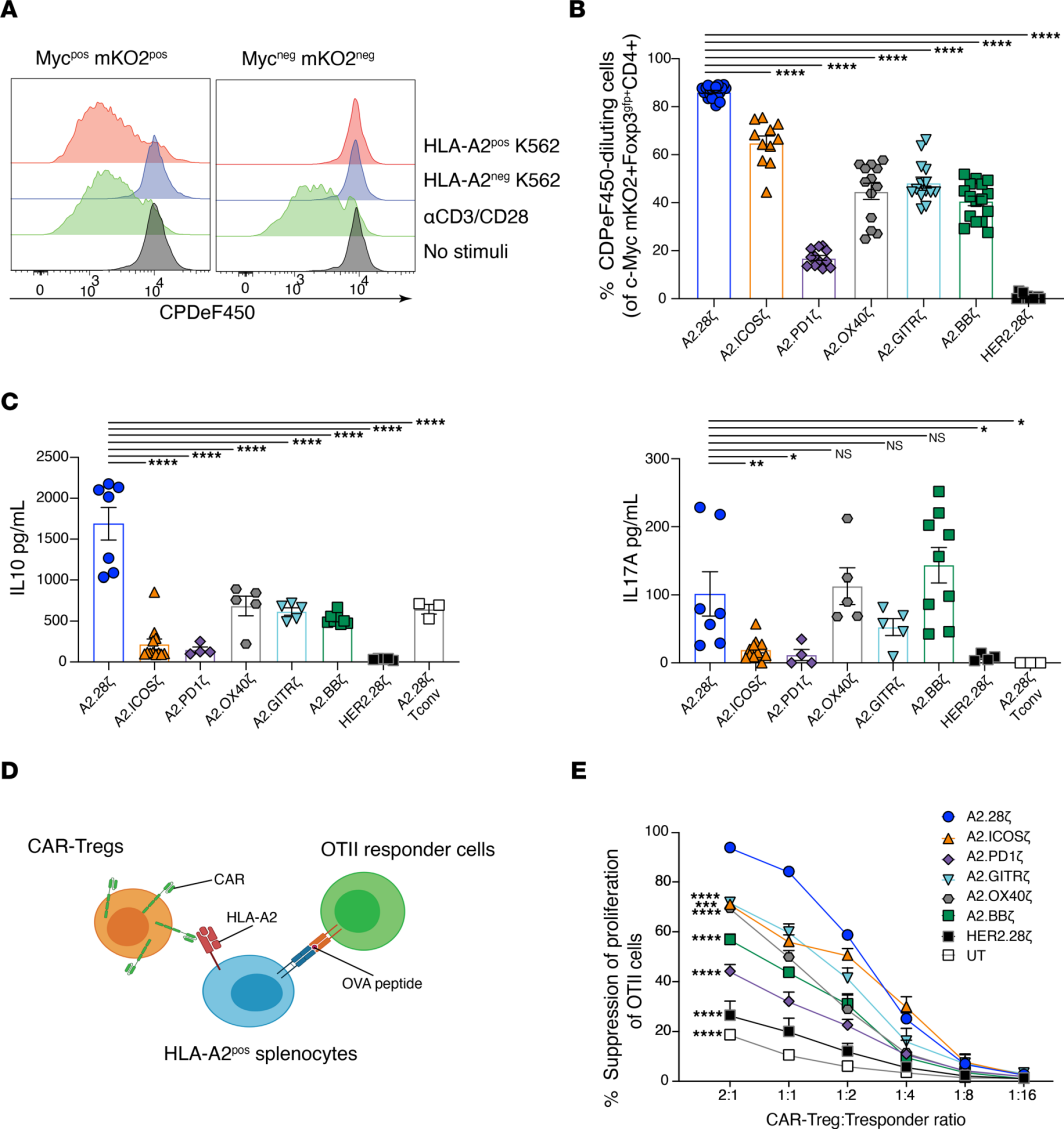
Costimulatory CAR variants differ in their ability to stimulate Tregs. (**A**–**C**) Tregs expressing the indicated CAR were stained with CPDe450 and cocultured with HLA-A2^+^ or HLA-A2^–^ K562 cells, polyclonal stimulated with anti-CD3/28, or left unstimulated for 3 days. (**A**) Representative histograms of at least 5 independent experiments comparing A2.28ζ CAR-Treg proliferation of gated CAR^+^ (c-Myc^+^mKO2^+^) or CAR^–^ (c-Myc^–^mKO2^–^) cells. (**B**) Frequencies of CAR-Tregs that divided after 3 days of coculture with HLA-A2^+^ K562s, determined by CPDeF450 dilution, gated on c-Myc^+^mKO2^+^Foxp3^gfp+^CD4^+^ cells; *n* = 11–20 replicates from at least 5 independent experiments. (**C**) Cytokine secretion after 3 days of coculture with HLA-A2^+^ K562s; *n* = 3–12 replicates from at least 3 independent experiments. (**D** and **E**) CAR-Tregs were cocultured with OTII CD4^+^ T cells at varying ratios in the presence of irradiated HLA-A2^+^ splenocytes and OVA peptide. (**D**) Schematic diagram of the linked suppression assay. (**E**) CAR-Treg–mediated suppression of the OTII CD4^+^ T cell proliferation, as determined by Ki67 expression; *n* = 3–6 replicates from at least 2 independent experiments. Tresponder, responder T cell; UT, untransduced. Data are reported as mean ± SEM. Statistical significance was determined using 1-way (**B** and **C**) or 2-way (**E**) ANOVA with a Holm-Šidak posttest comparing to CD28-based CAR-Tregs. *P < 0.05, **P < 0.01, ***P < 0.001, ****P < 0.0001.

To assess CAR variant function, CAR-Tregs were labelled with CPDeF450 and cocultured with K562 cells expressing HLA-A2 for 72 hours. Only Tregs expressing a CAR proliferated in response to HLA-A2 (Figure 2A). Differences in Treg proliferation were observed, with the CD28-encoding CAR inducing the strongest proliferative response, followed by the ICOS-encoding CAR (Figure 2B). TNFR family costimulatory CARs (OX40, GITR, and 4-1BB) induced a moderate proliferative response (Figure 2B), whereas the programmed cell death 1–encoding (PD-1–encoding) CAR induced little proliferation, corroborating our previous study in human Tregs (10) and other studies in CAR-T cells (49).

Analysis of cell culture supernatants revealed that Tregs expressing the CD28-encoding CAR secreted the highest levels of IL-10. CARs encoding TNFR family domains (OX40, GITR, 4-1BB) induced medium levels of IL-10, and PD-1– and ICOS-encoding CARs induced the lowest (Figure 2C, left). Low levels of IL-17A were secreted by Tregs expressing the ICOS- and PD-1–encoding CARs, in contrast to findings of a previous study performed with CAR-T cells that showed an ICOS-encoding CAR induced high IL-17A production (50) (Figure 2C, right). In comparison with A2-specific CAR-T cells, none of the CAR-Treg variants secreted significant amounts of pro-inflammatory cytokines or IL-2 (Supplemental Figure 3A).

To test how CAR signaling influenced Treg function, antigen-dependent, linked suppression assays were performed in which the ability of CAR-Tregs to inhibit OTII CD4^+^ T cell proliferation was measured (Figure 2D). Tregs expressing the CD28-based CAR exhibited the greatest suppressive function (Figure 2E and Supplemental Figure 3B). Tregs expressing the other CARs varied in their suppressive capacity. PD-1–encoding CAR-Tregs were the least suppressive but remained more suppressive than the polyclonal HER2-CAR or untransduced Treg controls. Thus, as we previously found in human CAR-Tregs (10), in an in vitro setting when CAR-Tregs are solely stimulated through the CARs, their activation and function are strongly influenced by the CAR-encoded costimulatory domain, with a clear superior effect of CD28.

### In vivo effects of Tregs expressing costimulatory CAR variants on skin rejection.

**Figure 3:**
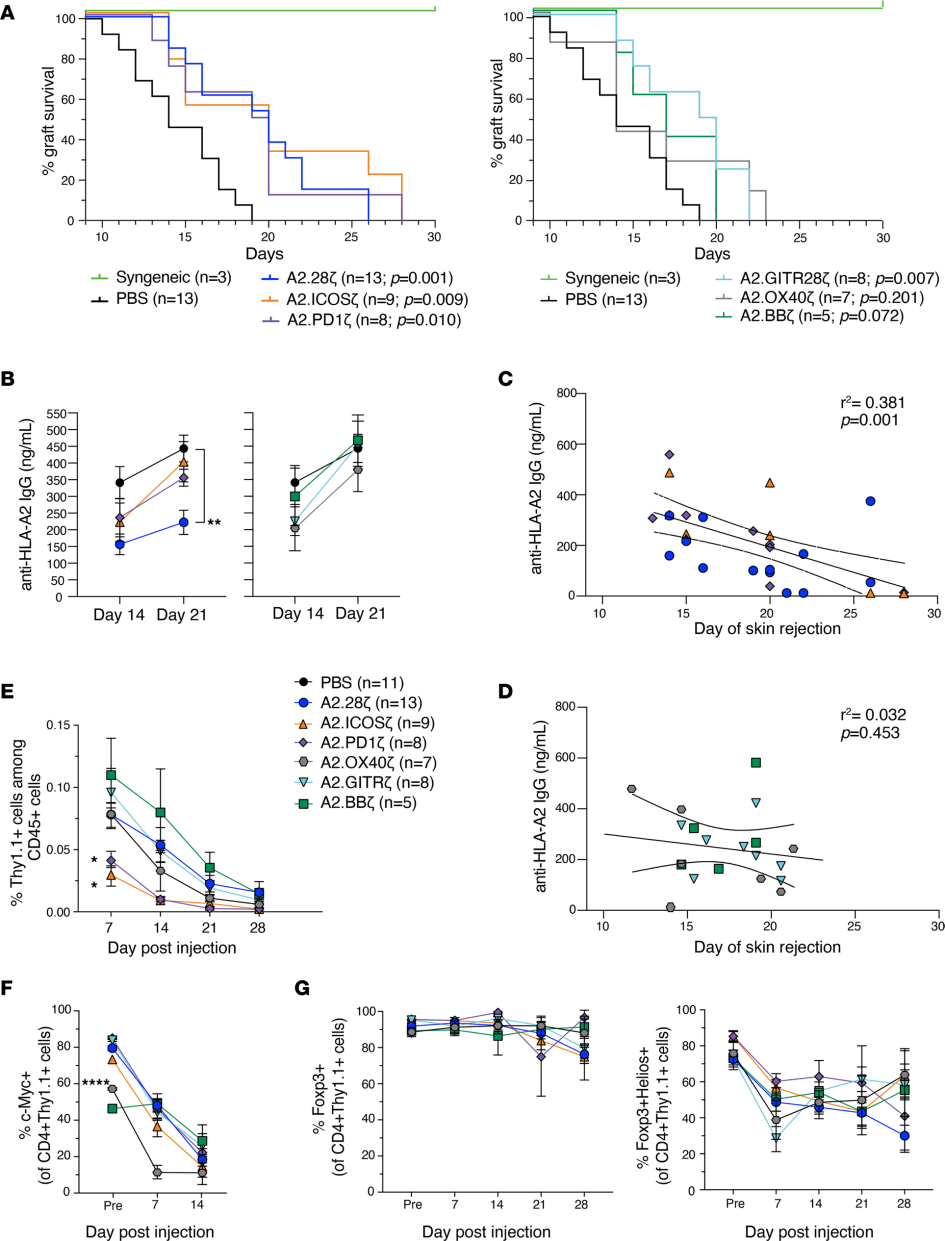
In vivo effects of Tregs expressing costimulatory CAR variants on skin rejection. BL/6 mice were transplanted with skin grafts from syngeneic or HLA-A2^+^ BL/6 mice and administered 1 × 10^6^ CAR-Tregs i.v. (**A**) Skin graft survival curves and (**B**) levels of anti–HLA-A2 IgG Abs from mice infused with Tregs expressing CARs encoding costimulatory domains from CD28 (left) or TNFR (right) family members. (**A**) Data from mice receiving no Treg treatment (PBS) or transplanted with syngeneic WT BL/6 grafts are shown in both graphs. (**C**) Correlation between anti–HLA-A2 IgG Abs in plasma at day 14 and skin graft rejection of mice receiving Tregs bearing CD28 family–based CARs. (**D**) Correlation between anti–HLA-A2 IgG Abs in plasma at day 14 and skin graft rejection of mice receiving Tregs bearing TNFR family–based CARs. (**E**) Persistence of CAR-Tregs measured as the percentage of Thy1.1^+^ CAR-Tregs of total CD45^+^ T cells in peripheral blood over time. (**F** and **G**) Phenotype of Thy1.1^+^CD4^+^ CAR-Tregs in peripheral blood over time including expression of (**F**) CAR (c-Myc^+^) and (**G**), FoxP3 alone (left), and FoxP3 with Helios (right). Data are reported as mean ± SEM pooled from 4 individual experiments with *n* = 3–13 mice per group. Statistical significance was determined using (**A**) log-rank Mantel-Cox test, (**B** and **E**–**G**) 2-way ANOVA with a Holm-Šidak posttest, and Pearson’s correlation (**C** and **D**). *P < 0.05, **P < 0.01, ****P < 0.0001.

We next compared the function of CAR-Treg variants using an immunocompetent mouse model of allogeneic skin transplantation (15). WT BL/6 mice received HLA-A2^+^ BL/6 skin grafts and were administered 1 × 10^6^ CAR-Tregs i.v. Consistent with our previous study findings (15), CAR-Tregs delayed but did not prevent skin rejection: median survival time was 20 days for mice treated with A2.CD28ζ CAR-Tregs versus 14 days for PBS (Figure 3A, left). CAR-Tregs encoding other CD28 family domains, ICOS or PD-1, also delayed skin rejection with median survival times of 20 days for ICOS and 19.5 days for PD-1. On the other hand, with the exception of GITR, Tregs encoding CARs with TNFR family–derived domains did not extend graft survival. The median survival times were 14 days for OX40-, 17 days for 4-1BB–, and 19.5 days for GITR-encoding CAR-Tregs (Figure 3A, right).

DSAs are important mediators of organ rejection (45), so CAR-Treg control of anti–HLA-A2 IgG generation was assessed. Mice treated with A2.CD28ζ CAR-Tregs had significantly lower levels of anti–HLA-A2 IgG compared with PBS mice (Figure 3B), corroborating our previous observations (15). Conversely, no other CAR-Treg we tested significantly reduced the levels of anti–HLA-A2 IgG compared to PBS mice. To assess if there was a correlation between control of anti–HLA-A2 IgG and graft rejection, a regression analysis was performed that revealed a negative correlation between amounts of anti–HLA-A2 IgG and graft survival (Supplemental Figure 4A). Interestingly, when this analysis was performed separately for CD28 and TNFR family–encoding CARs, the correlation was only present for the former (Figure 3, C and D).

CAR-Treg persistence and phenotype were tracked weekly in blood samples. Only Tregs expressing ICOS- or PD-1–encoding CARs persisted significantly less than A2.CD28ζ CAR-Tregs (Figure 3E and Supplemental Figure 4B). The waning of CAR-Treg persistence was not related to immunogenicity of the mKO2 transduction reporter, because CAR-Tregs with or without mKO2 expression displayed similar patterns of engraftment and persistence in blood and spleen (Supplemental Figure 4C). With the exception of the A2.OX40ζ CAR, there were no differences in the amount of CAR expression on Tregs in vivo (Figure 3F). Expression of Foxp3 and Helios were equivalent among all CAR-Treg groups, showing that none of the costimulatory domains negatively affected Treg stability in vivo (Figure 3G).

### A costimulatory domain is dispensable for CAR-Treg function in vivo.

**Figure 4:**
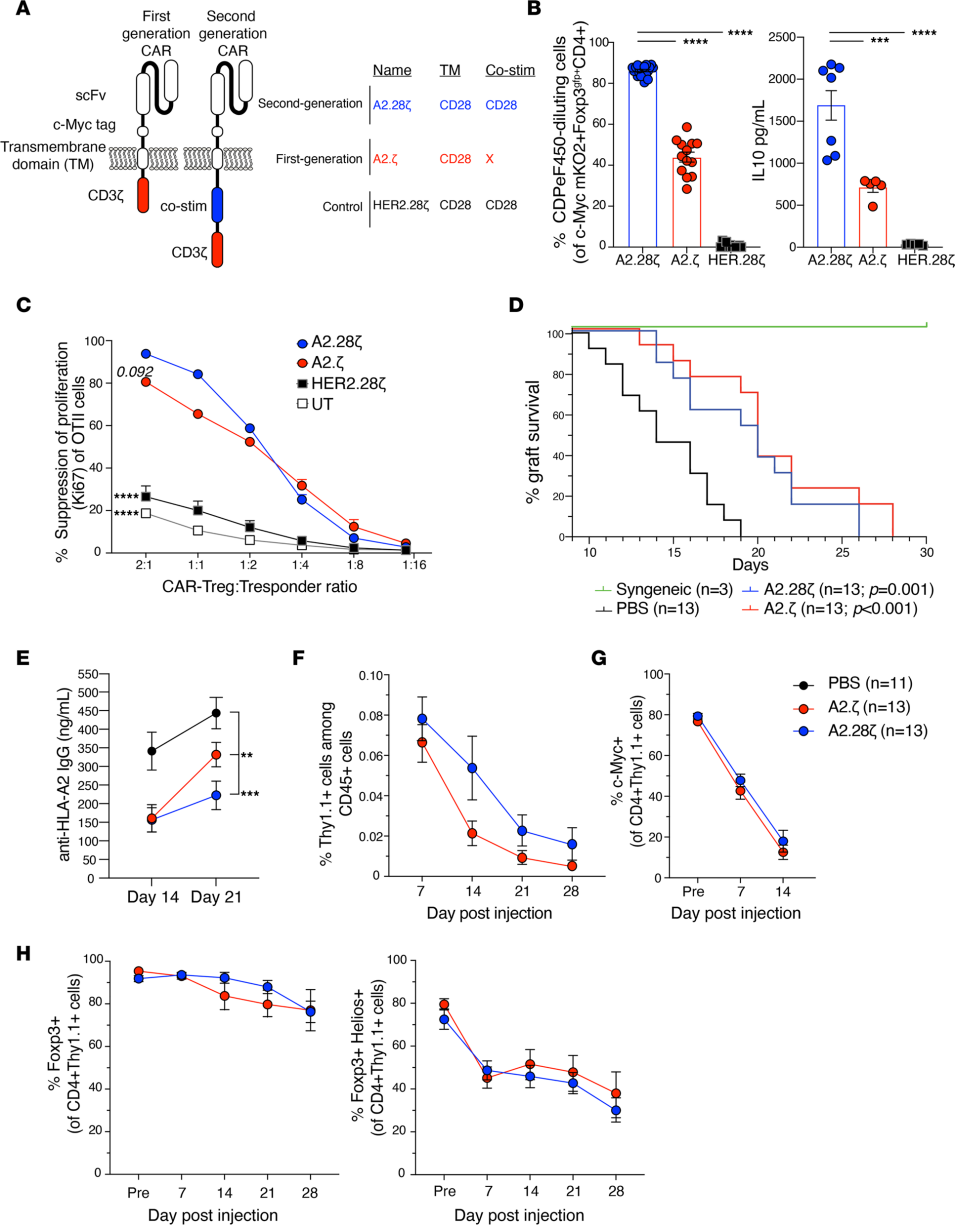
A CAR costimulatory domain is dispensable for CAR-Tregs in vivo. (**A**) Schematic diagram of the first- and second-generation CARs used. Tregs expressing the indicated CARs were stained with CPDe450 and cocultured with HLA-A2^+^ K562 cells for 3 days. (**B**) The percentage of CAR-Tregs that divided, determined by CPDeF450 dilution (left); *n* = 12–20 replicates from at least 5 independent experiments; and IL-10 secretion (right), measured in culture supernatants; *n* = 5–7 replicates from at least 3 independent experiments. (**C**) CAR-Tregs were cocultured with OTII CD4^+^ T cells at varying ratios in the presence of irradiated HLA-A2^+^ splenocytes and OVA peptide. CAR-Tregs mediated suppression of the OTII CD4^+^ T cell proliferation, as determined by Ki67 expression; *n* = 3–6 replicates from at least 2 independent experiments. UT, untransduced. (**D**–**H**) BL/6 mice were transplanted with skin grafts from syngeneic or HLA-A2^+^ BL/6 mice and administered 1 × 10^6^ CAR-Tregs i.v. (**D**) Skin graft survival curves and (**E**) levels of anti–HLA-A2 IgG Abs from mice infused with Tregs expressing first- and second-generation CARs. (**F**) Persistence of CAR-Tregs measured as the percentage of Thy1.1^+^ CAR-Tregs of total CD45^+^ T cells in peripheral blood over time. (**G** and **H**) CAR-Treg expression of (**G**) CAR (c-Myc+) and (**H**) FoxP3 and FoxP3 and Helios. In vivo data pooled from 3 individual experiments with *n* = 3–13 mice per group. Data are reported as mean ± SEM. Data from the A2.28z, HER2.28z, and UT conditions are also shown in Figure 2, C and E, and Figure 3, A, B, and E–G. Statistical significance was determined using (**B**) 1-way or (**C** and **E**–**H**) 2-way ANOVA with a (**C** and **E**) Holm-Šidak posttest or (**D**) log-rank Mantel-Cox test. **P < 0.01, ***P < 0.001, ****P < 0.0001. Co-stim, costimulatory; UT, untransduced.

The minimal differences between some CAR-Treg variants in this immunocompetent setting contrast with the in vitro data generated in this study and also with previous studies that used immunodeficient mouse models, both of which clearly showed a superior function of CD28-encoding CARs (10, 11, 16, 51). To understand the mechanistic basis for these findings, we hypothesized that, in an immunocompetent in vivo setting, CAR-Tregs may receive additional costimulatory signals that compensate for weaker CAR-mediated activation. To address this possibility, we tested a first-generation (A2.ζ) CAR that lacked a costimulatory domain (Figure 4A) and performed direct comparisons with the second-generation A2.28ζ CAR. The A2.ζ and A2.28ζ CARs were expressed at similar levels and no differences in Foxp3^gfp^ expression were observed (Supplemental Figure 5A). In vitro assays revealed that upon stimulation with K562 cells only expressing HLA-A2, A2.ζ CAR-Tregs had significantly less proliferation and lower cytokine secretion levels than did A2.28ζ CAR-Tregs (Figure 4B and Supplemental Figure 5B). When tested with the OTII linked suppression assay, there was a trend toward lower antigen-specific suppression with A2.ζ compared with A2.28ζ CAR-Tregs (Figure 4C and Supplemental Figure 5C).

After adoptive transfer into an immunocompetent skin transplant model, A2.ζ and A2.28ζ CAR-Tregs were equal in their ability to delay skin rejection (median survival time was 20 days for both versus 14 days for PBS mice) (Figure 4D). Additionally, A2.ζ and A2.28ζ CAR-Tregs were similarly able to suppress the generation of anti–HLA-A2 IgG Abs (Figure 4E) and levels of anti–HLA-A2 IgG were correlated with graft rejection (Supplemental Figure 6A). There was a trend, although it was not significant, toward lower persistence of A2.ζ CAR-Tregs in peripheral blood (Figure 4F and Supplemental Figure 6B), and there was no difference in functional markers (Supplemental Figure 6C). There was also no difference in A2.ζ and A2.28ζ CAR-Treg numbers in draining lymphoid node (dLN) or spleen (Supplemental Figure 7, A and B). A2.ζ and A2.28ζ CAR-Tregs also did not differ in expression of the CAR (Figure 4G and Supplemental Figure 7C), Foxp3, and Helios (Figure 4H and Supplemental Figure 7, D and E). Together, these results suggest CAR-encoded costimulation is redundant for CAR-Treg function in an immunocompetent model.

### CAR-Tregs integrate exogenous and CAR-encoded costimulation in vitro and in vivo.

**Figure 5:**
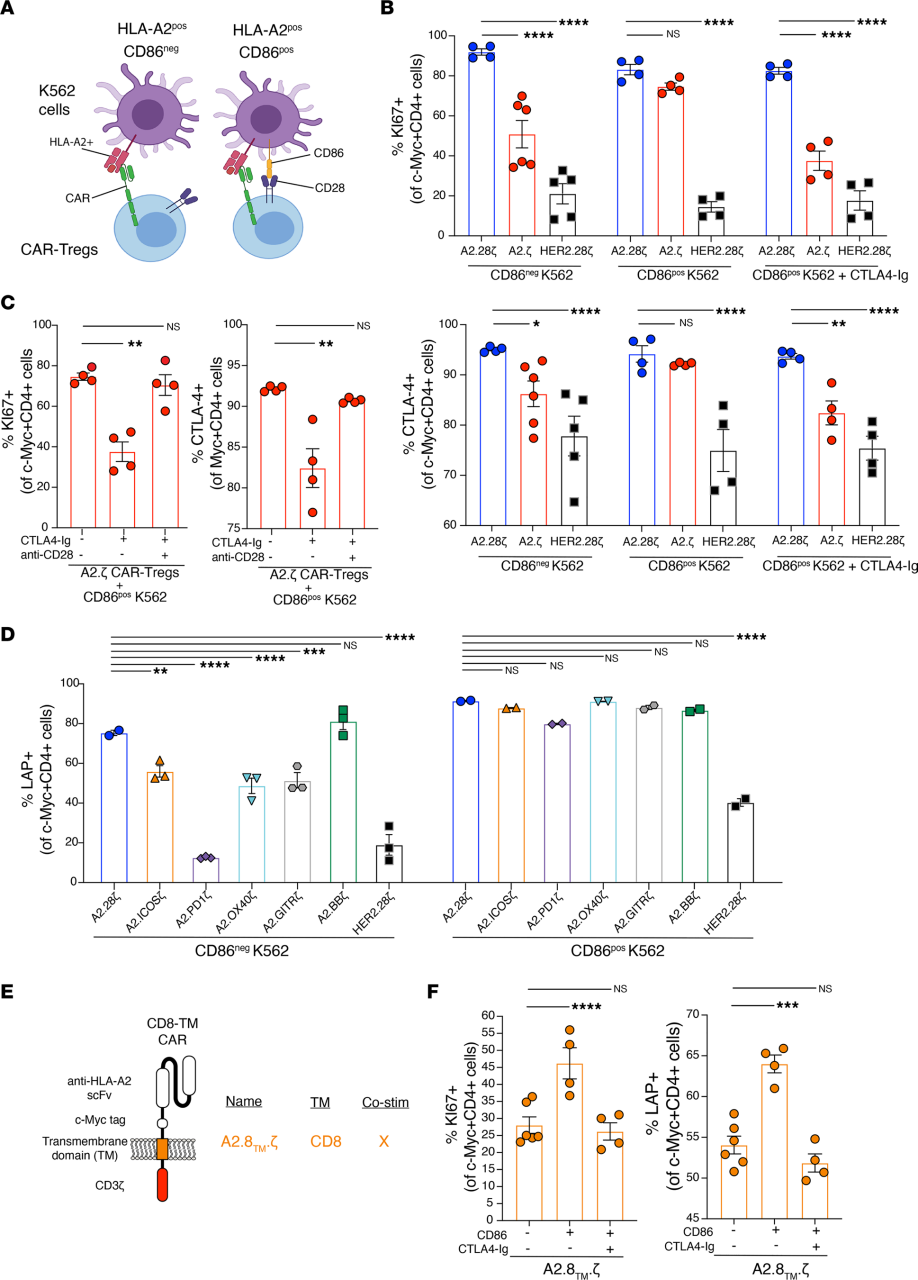
Effect of exogenous costimulation on CAR-Tregs. CAR-Tregs were co-cultured with CD86^pos^HLA-A2^pos^ or CD86^neg^HLA-A2^pos^ K562 cells at a 1:2 K562/Tregs ratio for 3 days. (**A**) Schematic diagram of assay. (**B** and **C**) Ki67 and CTLA-4 expression in CAR-Tregs following 3 days of co-culture, gated on c-Myc^+^CD4^+^ live cells; *n* = 4 to 6 replicates from 2 independent experiments. (**D**) LAP expression in different costimulatory-encoding CAR-Tregs following 3-days of co-culture, gated on c-Myc^+^CD4^+^ live cells; *n* = 2 to 3 replicates from 1 experiment. (**E**) Schematic diagram of the CD8α-TM CARs generated. (**F**) Ki67 and LAP expression in first-generation CD8α-TM CAR-Tregs following 3 days of co-culture, gated on c-Myc^+^CD4^+^ live cells; *n* = 4 to 6 replicates from 1 experiment. Where indicated, CTLA-4–Ig and an anti-CD28 agonist mAb were added at 10 μg/mL. Data are reported as mean ± SEM. Statistical significance was determined using 1-way (**C** and **F**) or 2-way (**B** and **D**) ANOVA with a Holm-Šidak posttest. *P < 0.05, **P < 0.01, ***P < 0.001, ****P < 0.0001. Co-stim, costimulatory.

A fundamental difference between our in vivo studies and those previously performed with humanized mice is that the latter lacks professional APCs. As such, we hypothesized that in an immunocompetent in vivo setting, CD28 naturally expressed by the CAR-Tregs may engage CD80/86 on APCs and compensate for a weak or absent CAR-encoded costimulatory signal. To investigate this possibility, A2.ζ or CD28-containing second-generation (A2.28ζ) CAR-Tregs were stimulated with HLA-A2^+^CD86^–^ or HLA-A2^+^CD86^+^ K562s, after which proliferation and activation were determined by the expression of Ki67, CTLA-4, PD-1, and latency-associated protein (LAP) (Figure 5A). In the absence of exogenous CD86, A2.28ζ CAR-Tregs were significantly more activated and proliferative than A2.ζ CAR-Tregs (Figure 5B and Supplemental Figure 8A). However, in the presence of CD86, these differences diminished, with the first- and second-generation CAR-Tregs showing similar levels of activation and proliferation (Figure 5B and Supplemental Figure 8A). To further validate these findings, Tregs were stimulated with HLA-A2^+^CD86^+^ K562s in the presence of CTLA-4–Ig to block CD86. CTLA-4–Ig treatment reduced the proliferation and activation of A2.ζ CAR-Tregs to similar levels found in absence of CD86 (Figure 5, B and C, and Supplemental Figure 8, A and B). The inhibitory effect of CTLA-Ig was overcome by adding an agonistic anti-CD28 mAb able to induce CD28 signaling independently of CD86 (Figure 5C and Supplemental Figure 8B).

Having shown that costimulation through native CD28 can act in conjunction with CAR-mediated CD3ζ signaling to fully activate Tregs, we next asked how CD28 signaling combines with signals from other costimulatory domain CARs. Corroborating our previous findings, Tregs encoding different CD28 or TNFR family CARs were activated to differing degrees upon coculture with HLA-A2^+^CD86^–^ K562s. However, these differences were reduced in the presence of HLA-A2^+^CD86^+^ K562s, demonstrating that the function of CAR-Tregs is influenced by both CAR-dependent and CAR-independent stimulation (Figure 5D and Supplemental Figure 9A).

It has been shown that CARs encoding a CD28-derived TM domain can dimerize with endogenous CD28 (52). To exclude the possibility that our observations could be related to interactions between the CAR CD28 TM and native CD28, resulting in the presence of a CD28 signal even in the absence of a CAR-encoded CD28 endodomain, we generated new CARs encoding a CD8α-derived TM (Figure 5E). Tregs expressing the indicated CARs were stimulated with HLA-A2^+^CD86^–^ or HLA-A2^+^CD86^+^ K562 cells, revealing that first-generation CARs with CD8α TM domains are similarly able to respond to exogenous CD28 stimulation (Figure 5F and Supplemental Figure 9B), suggesting a minimal impact of the type of TM in this process.

**Figure 6:**
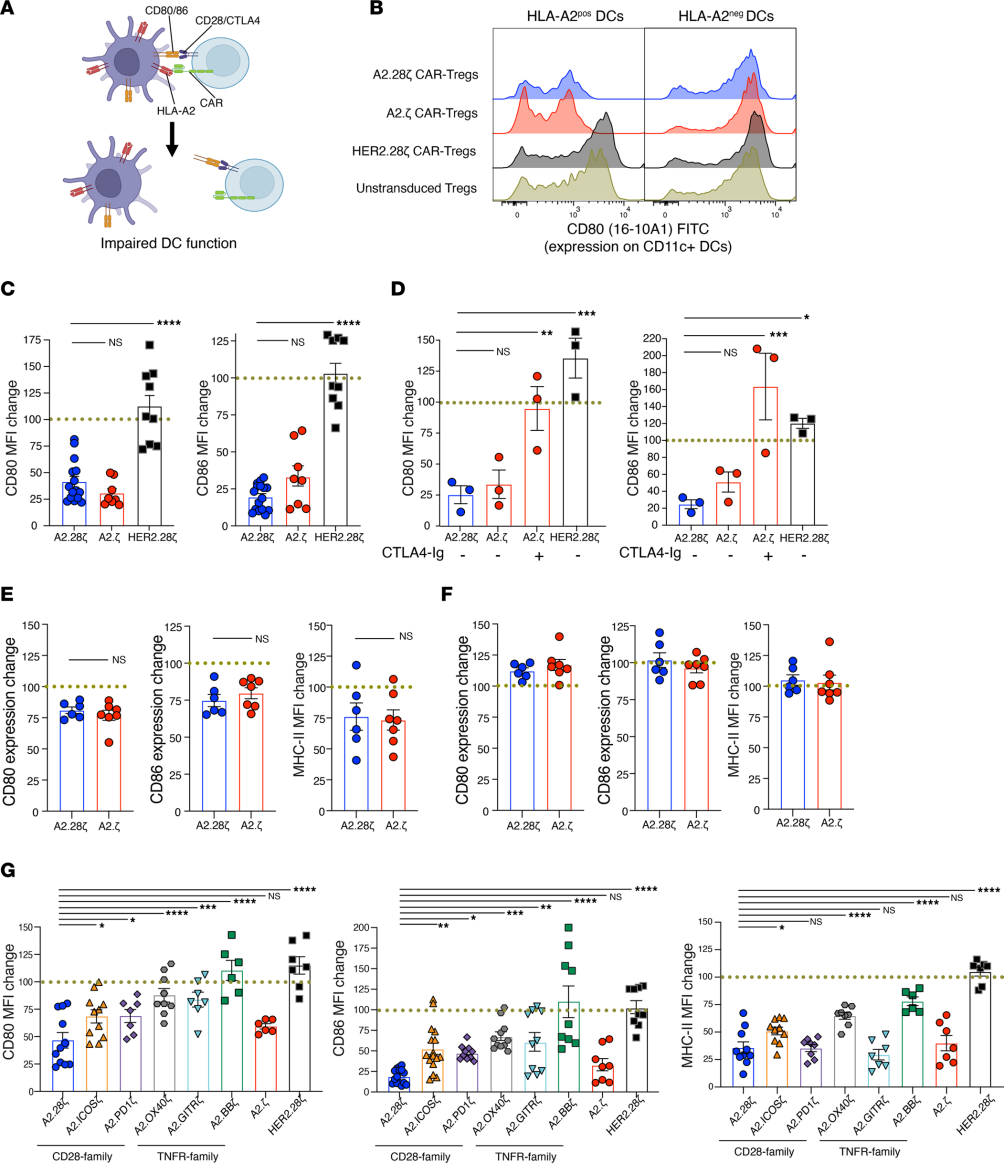
In vivo and in vitro APC suppression by first- and second-generation CAR-Tregs. (**A**–**D** and **G**) For in vitro assays, CAR-Tregs were cocultured with splenic HLA-A2^+^ CD11c^+^ DCs at a ratio of 1:2 or 1:5 DCs to Tregs for 1 or 2 days. (**A**) Schematic of in vitro DC suppression assay. (**B**) Representative histograms of at least 5 independent experiments showing CD80 expression on CD11c^+^ DCs after 2-days of culture with the indicated types of Tregs. (**C**) Expression of CD80 (left) and CD86 (right) in HLA-A2^+^ CD11c^+^ DCs relative to DCs cultured with untransduced Tregs (dotted line); *n* = 8–15 replicates from at least 5 independent experiments. (**D**) In vitro DC suppression assays performed with or without 10 μg/mL CTLA-4–Ig; *n* = 3 replicates from 2 independent experiments. (**E** and **F**) For in vivo assays, BL/6 mice were transplanted with skin grafts from HLA-A2^+^ BL/6 mice and treated or not with 1 × 10^6^ CAR-Tregs. dLN and spleen tissues were collected at day 7 after CAR-Treg infusion; *n* = 6–7 mice per group from 2 independent experiments. (**E**) Expression of CD80, CD86, and MHC-II in DCs from dLNs of mice treated with CAR-Tregs relative to untreated mice (dotted lines; average of 3–4 untreated mice per each experiment). (**F**) Expression of CD80, CD86, and MHC-II in DCs from spleens of mice treated with CAR-Tregs relative to untreated mice (dotted lines; average of 3–4 untreated mice per each experiment). (**G**) Expression of CD80 at day 1 (left), CD86 at day 2 (center), and MHC-II at day 1 (right) on HLA-A2^+^CD11c^+^ DCs treated with different types of CAR-Tregs in vitro; *n* = 6–16 replicates from 4 (CD80/MHC-II) or 5 (CD86) independent experiments. (**C**, **D**, and **G**) Data are shown relative to DCs cultured with untransduced Tregs, which were normalized to 100% (dotted lines). (**E** and **F**) Data are shown relative to the expression of CD80, CD86, and MHC-II in DCs from nontreated mice, which were normalized to 100% (dotted lines). Data are reported as mean ± SEM. Statistical significance was determined using (**C**, **D**, and **G**) 1-way ANOVA with a Holm-Šidak posttest and (**E** and **F**) Student’s *t* test. *P < 0.05, **P < 0.01, ***P < 0.001, ****P < 0.0001.

To further confirm that CAR-encoded costimulatory domains are dispensable for Tregs if costimulation is provided by natural APCs (i.e., rather than K562 cells), we analyzed the ability of A2.28ζ and A2.ζ CAR-Tregs to inhibit the antigen-presenting capacity of DCs. CAR-Tregs were cocultured with HLA-A2^+^CD11c^+^ DCs for 24–48 hours, after which the expression of CD80, CD86, and MHC class-II in the DCs was assessed (Figure 6, A and B). A2.28ζ and A2.ζ CAR-Tregs were equally able to suppress CD80, CD86 (Figure 6C), and MHC-II expression (Supplemental Figure 10A). This effect was consistent at different time points and CAR-Treg to DC ratios (Supplemental Figure 10B). In concordance with previous results, the in vitro suppressive effect of A2.ζ CAR-Tregs was strongly inhibited by CTLA-4–Ig (Figure 6D).

To ask if A2.28ζ and A2.ζ CAR-Tregs can also have similar effects on DCs in vivo, we analyzed the expression of CD80, CD86, and MHC-II on DCs from dLNs and spleens of mice with A2^+^ skin transplants that were or were not treated with A2.28ζ or A2.ζ CAR-Tregs (Supplemental Figure 11A). Compared with DCs of nontreated mice (dotted line in Figure 6, E and F), treatment with either A2.28ζ or A2.ζ CAR-Tregs caused a similar reduction of CD80, CD86, and MHC-II expression in DCs from dLNs (Figure 6E) but had no effect on splenic DCs (Figure 6F).

Finally, to further investigate if interactions between CAR-Tregs and DCs could contribute to the differential in vivo function for all the CAR variants tested in this study, we conducted DC suppression assays with other CD28 and TNFR family CAR-Tregs. A2.28ζ and A2.ζ CAR-Tregs mediated the highest suppression of CD80, CD86, and MHC-II on DCs, followed by A2.ICOSζ and A2.PD1ζ CAR-Tregs (Figure 6G). A2.GITRζ CAR-Tregs showed an intermediate effect, whereas A2.OX40ζ and A2.BBζ CAR-Tregs showed poor suppression (Figure 6G). These data mirror the in vivo skin graft survival results and further confirm our previous finding (10) that in vitro suppression of DCs strongly correlates with in vivo Treg function.

## Discussion

Understanding how the structure of a CAR affects Treg function is critical to guide their clinical implementation. Here, we studied how different CAR costimulatory domains affect Treg function in an immunocompetent mouse model of skin transplantation. Although 4-1BB– and OX40-encoding CAR-Tregs did not have a significant therapeutic effect, CD28-, ICOS-, PD-1–, and GITR-encoding CAR-Tregs were similarly efficacious in vivo. Further comparisons between Tregs expressing a first- (A2.ζ) or second- (A2.28ζ) generation CAR revealed equivalent function, leading us to study a possible role for costimulation via the native CD28 receptor. These studies showed that native CD28 signaling can compensate for a lack of CAR-mediated costimulation, providing a significant advance in our understanding of how CAR-Tregs interact with host immune cells and regulate alloimmunity.

We and others, using immunodeficient mouse models, previously compared the function of CARs encoding different costimulatory domains in Tregs and found that a CD28 costimulatory domain was optimal for Treg potency, stability, persistence, and in vivo function (10, 11, 16, 51). CARs carrying alternative costimulatory domains had limited in vitro and in vivo function (10, 11, 16, 51). In contrast, in the immunocompetent transplant setting used here, Tregs expressing CARs encoding costimulatory domains from ICOS, PD-1, or GITR were similar to CD28 in terms of protection from skin rejection, although notably not control of DSA generation. Our data suggest that, at least for some CARs, this could be related to the combination of native CD28 and CAR-mediated signaling, with the former compensating for lower CAR-mediated activation.

CD28 is a major costimulatory receptor for Tregs (53–56), but these cells express a variety of costimulatory molecules (48, 57) that have been reported to have positive, negative, or mixed effects (48, 58). Thus, it is possible that certain combinations of costimulatory signaling driven by the CAR and/or natural coreceptors could be harmful and cause Treg dysfunction (51). For example, OX40 signaling helps maintain Treg homeostasis (59) but also inhibits their suppressive function and reduces Foxp3 expression (60, 61). In previous studies using immunodeficient mouse models, CARs carrying costimulatory domains from TNFR family members, such as 4-1BB, showed no therapeutic protection (10, 11, 16, 51) and were associated with exhaustion (51) and loss of Treg stability (10, 51). Similarly, agonist Abs binding TNFR family receptors such as 4-1BB or OX40 enhance antitumor immunity by depleting Tregs (62) or blocking their suppressive function (63). Supporting these studies, we found that Tregs expressing 4-1BB– or OX40-containing CARs did not efficiently suppress DCs even when natural costimulation was available. Importantly, the suppressive effect of 4-1BB– and OX40-encoding CAR-Tregs on DCs was even lower than that of first-generation CAR-Tregs, which lack any costimulatory domain in their CAR, demonstrating a dominant-negative role of these TNFR family costimulatory domains on Treg function. Our in vivo data showing that Tregs with CARs encoding 4-1BB and OX40 costimulatory domains have no protection from graft rejection or control of DSA generation are also consistent with these findings.

The functional effect of CAR costimulatory domains can be influenced by other factors. For example, negative effects of OX40 on Tregs could be modulated by IL-2 (59), and inhibition of mTOR signaling prevents some of the negative effects of 4-1BB signaling in CAR-Tregs (51). In our immunocompetent transplant setting, no signs of tonic signaling or loss of Foxp3 or Helios expression were observed in 4-1BB–based CAR-Tregs even 4 weeks after adoptive transfer; at the same time, however, no in vivo protection was observed. Thus, in vivo costimulation from the native CD28 receptor or other receptors might partially overcome some deleterious effects of these otherwise harmful costimulatory signals (64) but does not restore their function.

CARs were originally developed for use in cancer with the goal of directing T cells to kill tumor cells (65). These tumor cells often overexpress coinhibitory receptors as an immune escape mechanism and may not express costimulatory molecules such as CD80 or CD86 (66, 67). As such, first-generation CARs lacking costimulation showed low persistence and modest clinical outcomes in a cancer setting (68–70), and the provision of costimulation, mainly CD28 (71) or 4-1BB (50), in a second-generation CAR format greatly increased their persistence and clinical success (72). Distinct from CAR T cells, the inclusion of neither a CD28 nor 4-1BB costimulatory domain improved in vivo persistence compared with first-generation CAR-Tregs in immunocompetent mice. In Tregs, studies of first-generation CARs are limited, with only 1 study in immunodeficient mice showing little protection from xenoGvHD (10). Conversely, we found that in an immunocompetent mouse setting, first- and second-generation CAR-Tregs offer the same protection from rejection and control of DSA generation.

APCs naturally confer costimulation and maturation signals to Tregs and are a major target for Treg suppression (47). In the context of transplantation, interactions between Tregs and APCs in graft-surrounding areas are important for controlling alloimmunity and inducing tolerance (73). Our data suggest that first-generation CAR-Tregs could receive natural costimulation via interaction with these cells. This possibility is supported by our in vitro data on Tregs that show native CD28 costimulation compensates for absent CAR-encoded costimulation. Similar findings were reported with CAR-T cells in vitro (74, 75): if costimulatory molecules are provided, first- and second-generation CARs equivalently activate T cells. Importantly, we found that first- and second-generation CAR-Tregs were similarly able to suppress the expression of costimulatory molecules on DCs in vitro but also in vivo, with reduced in vivo expression of CD80, CD86, and MHC-II expression in DCs compared with untreated mice. Collectively, these data highlight that, in vivo, CAR-Treg function is ultimately determined by an integrated response to CAR- and native costimulation-mediated signaling, which is naturally mediated by their interaction with APCs.

An outstanding question is where CAR-Tregs would encounter donor antigen and costimulation. Skin-resident APCs play an important role in the regulation of alloimmunity (76–79), and in our immunocompetent skin graft model, these cells could deliver both CAR and costimulatory signals to CAR-Tregs. After activation in the allograft, Treg migration to dLNs is thought to be essential for establishing immune tolerance (80). Accordingly, A2.ζ and A2.28ζ CAR-Tregs accumulated similarly in dLNs. This suggests that A2.ζ and A2.28ζ CAR-Tregs are equivalently activated in the allograft’s surrounding areas, which is in line with the lack of differences in activation or proliferation markers observed in peripheral blood. Skin donor APCs also migrate to surrounding lymphoid nodes (81), and/or host APCs could be cross-dressed with HLA-A2 via exosome-mediated mechanisms (82–84), which could also confer CAR-mediated stimulation to CAR-Tregs migrating to these anatomical structures. The fact that first- and CD28-based second-generation CAR-Tregs were both able to similarly control DSA generation and influence costimulatory molecule expression in DCs from dLNs suggests that CAR-Tregs migrating to dLNs not only receive costimulation but also CAR signals. The lack of effect of CAR-Tregs on costimulatory molecule expression in DCs from other anatomical structures like spleen not only supports this possibility but also points out that CAR-Treg–mediated tolerance could be anatomically restricted to the allograft’s surrounding areas.

Notably, CAR-Treg therapy delayed graft rejection but did not induce indefinite graft survival. Similar results have been reported by previous studies using different models of transplantation (8, 9). A potential reason for this could be the inability of CAR-Tregs to control the high numbers of alloreactive T cells generated after transplantation, an issue that can be resolved by administrating cytotoxic or immunosuppressive preconditioning treatments before infusing Tregs (85, 86). Another reason could be the high stringency of immunocompetent mouse models of transplantation, particularly of the skin allograft model. Studies exploring the use of A2-specific CAR-Tregs alone in a single HLA-A2–mismatched heart transplant model also did not induce long-term tolerance but did extend graft protection longer than our skin allograft model did (17). The shorter protection observed in our skin transplant models versus heart or other transplant models (17) could be related to the lack of vascularization, which could hinder the ingress of CAR-Tregs to the graft. However, skin allograft models facilitate testing of multiple CAR-Treg groups in parallel, which is not feasible with other less stringent transplant models, due to their complexity.

Another potential limiting factor for CAR-Tregs is their short in vivo persistence, which could be driven by multiple mechanisms. Our data show that immunogenicity of the mKO2 transduction reporter does not affect persistence, and in another recent study, researchers did not observe any further benefits of higher doses of CAR-Tregs in a cardiac transplant model (17). Because CAR-Tregs uptake HLA-A2 molecules by trogocytosis (15), it is possible that they become targets for anti–HLA-A2 Abs and are depleted. Low levels of IL-2 could also affect CAR-Treg persistence (87), as could diminishing levels of the target antigen, which may become limiting as rejection progresses. Overall, investigation of strategies to enhance persistence, such as by repeated dosing, or coadministration of adjunct therapies such as IL-2, is warranted.

TM domains have been described to have a role in CAR stability and function (9). We and others previously explored how different TM domains affect the expression and function of CARs carrying different costimulatory domains (9, 10, 50). In this study, we designed different CAR variants including TM domains previously described to increase the stability and expression of different costimulatory domain CARs in Tregs (10). Thus, the effects of the studied CARs shown here could be interpreted as a conjunction of both TMs and costimulatory domains.

Overall, our results contribute to the understanding of how alternative costimulatory domains affect the in vivo function of CAR-Tregs and demonstrate that CAR-mediated costimulation is not essential for in vivo function of Tregs. These data provide an important step forward in our understanding of the biology of CAR-Tregs and how to best optimize them for clinical applications.

## Methods

### Generation of signaling domain CAR variants.

CAR variants were generated by replacing the TM and costimulatory domains of a previously characterized A2-specific CAR (10, 15). Domain sequences were obtained from UniProt and codon optimized for mouse (Supplemental Table 1). The resulting CARs encoded an A2-specific scFv (12), a CD8α-derived hinge, a c-Myc epitope tag, the indicated TM and costimulatory domains, and CD3ζ. A HER2-specific CAR served as an antigen-nonspecific negative control (10, 15). CARs were subcloned into a murine stem cell virus–based vector upstream of an internal ribosome entry site–monomeric Kusabira-Orange 2 (mKO2) reporter, and retroviral particles were generated as described previously (15).

### Animals.

BL/6, BL/6-Foxp3^gfp^ × Thy1.1 BL/6, HLA-A2^+^ BL/6 mice [B6.Cg-Tg(HLA-A/H2-D)2Enge/J], and OTII BL/6 mice [B6.Cg-Tg(TcraTcrb)425Cbn/J] were purchased from Jackson Laboratory and bred in-house under specific pathogen–free conditions.

### CAR-Treg generation.

CAR-Tregs were generated as described previously (15, 88). Briefly, lymph nodes and spleen from female or male C57BL/6-Foxp3^gfp^ × Thy1.1 mice 16–24 weeks old were collected and CD4^+^ T cells were isolated by negative selection (STEMCELL Technologies). Tregs were sorted as live CD4^+^CD8^–^Thy1.1^+^Foxp3^gfp+^ using an MoFlo Astrios cell sorter (Beckman Coulter) (Supplemental Figure 1A), stimulated with anti-CD3/CD28 Dynabeads (Thermo Fisher Scientific), expanded in the presence of recombinant human IL-2 (1,000 U/mL; Proleukin) and rapamycin (50 nmol/L; Sigma-Aldrich), and transduced after 2 days. Dynabeads were removed on day 7 and cells were rested overnight in 1,000 U/mL, or 100 U/mL IL-2 for 2 days before use for in vivo or in vitro assays, respectively. CAR expression and Treg purity were determined after expansion (Supplemental Figure 1B).

### Proliferation, activation, and cytokine production.

CAR-Tregs were labelled with CPDeFluor450 proliferation dye (5 μM; eBioscience), then stimulated with irradiated (125 Gy) HLA-A2^+^CD86^–^, HLA-A2^+^CD86^+^, or HLA-A2^–^CD86^–^ K562 cells at a 1:2 K562 to Treg ratio with 100 U/mL IL-2. After 72 hours, activation markers and proliferation (CPDe450 dilution or Ki67 expression) were assessed by flow cytometry, and cell culture supernatants were collected to measure cytokine secretion, using a cytometric bead array (BD Biosciences). Where stated, 10 μg/mL CTLA-4–Ig (Orencia) and/or an anti-CD28 agonist Ab (clone: 37.51; BD Biosciences).

### Suppression assays.

For T cell suppression, responder CD4^+^ T cells were isolated from OTII BL/6 mice by negative selection (STEMCELL Technologies). Splenocytes from WT or HLA-A2^+^ BL/6 mice were depleted of Thy1.2^+^ cells (STEMCELL Technologies), irradiated (20 Gy), and 175,000 splenocytes were cocultured in a U-bottom, 96-well plate with 25,000 OTII T cells with 200 ng/mL OVA_323–339_ peptide (Sigma-Aldrich) and varying ratios of Tregs in a volume of 200 mL. OTII proliferation was measured by flow cytometry after 4 days, and percentage suppression was calculated as the inhibition of responder T cell proliferation relative to responder T cells cultured without Tregs.

For in vitro DC suppression, splenic CD11c^+^ DCs were isolated by positive selection (STEMCELL Technologies) from WT or HLA-A2^+^ BL/6 mice and cultured with CAR-Tregs (DC to Treg ratio 1:2 or 1:5). Suppressive effects of CAR-Tregs were measured as the percentage of decreased expression of costimulatory (CD80 and CD86) and MHC-II molecules on DCs after 1 and/or 2 days.

### Skin transplantation.

Female and male WT C57BL/6 mice, 10–14 weeks old, were transplanted with dorsal skin grafts from sex-matched, WT or HLA-A2^+^ BL/6 mice. Where stated, mice were injected with 1 × 10^6^ CAR-Tregs (equivalent to 30 × 10^6^/kg to 50 × 10^6^/kg) into the tail vein at the time of transplantation (15). For immunogenicity study, 5 × 10^5^ mKO2^+^ and 5 × 10^5^ mKO2^–^ CAR-Tregs were coinjected. Grafts were covered with a petroleum jelly gauze patch and wrapped with CoFlex bandage (Nexcare; 3M). Bandages were removed after 10 days and grafts monitored for rejection until 30 days after transplantation. Graft rejection was defined as described previously (15).

To track CAR-Tregs in tissue and their in vivo effect on DC expression of costimulatory and MHC-II molecules, spleen and dLN tissues were collected at day 7 after surgery, mechanically disaggregated, and studied by flow cytometry. In dLNs, DCs were defined as live CD45^+^, Ly6G^–^, SiglecF^–^, CD3e^–^, CD19^–^, CD11c^+^ MHC^+^ (Supplemental Figure 11B). Splenic DCs were defined as live CD45^+^, Ly6G^–^, SiglecF^–^, CD3e^–^, CD19^–^, F4/80^–^, CD11c^+^ MHC^+^ (Supplemental Figure 11C). For immunogenicity study, mKO2^+^ and mKO2^–^ CAR-Treg relative frequencies were tracked in peripheral blood and spleen from samples collected weekly. RBCs were lysed using ammonium chloride, and Fc receptors were blocked using anti–mouse CD16/CD32 (BD Biosciences) before staining.

### Anti–HLA-A2 IgG quantification.

Anti–HLA-A2 IgG titers were determined using a cell-based ELISA. HLA-A2^+^ K562 and control K562 cells were seeded in a 96-well plate and blocked with rat serum (STEMCELL Technologies) for 30 minutes at room temperature (RT). Plasma samples were added (1:800 dilution) and incubated for 1 hour at RT. A goat anti–mouse IgG APC secondary Ab (Invitrogen) was added (1:700 dilution) and incubated for 1 hour at RT. A standard curve was made using purified anti–HLA-A2 Ab (clone: BB7.2; BD Biosciences). Cells were analyzed by flow cytometry and concentration was calculated on the basis of MFI using a 4PL curve.

### Flow cytometry.

Flow cytometry was performed in adherence to Cossarizza et al. (89). Flow cytometric Abs are shown in Supplemental Table 2. Cells were extracellularly stained in the presence of the fixable viability dye eFluor 780 (Thermo Fisher Scientific) to exclude dead cells. Staining for intracellular markers was performed using the Foxp3/Transcription Factor Staining Buffer Set (Thermo Fisher Scientific). Data were acquired using an LSR Fortessa II, A5 Symphony (BD Biosciences), or CytoFLEX (Beckman Coulter) cell analyzer and analyzed using FlowJo, version 10.7.1 (Tree Star).

### Statistics.

Data were analyzed using GraphPad Prism 9.3.1 and are reported as mean ± SEM. Statistical significance was determined using Pearson’s correlation, Student’s *t* test (2 tailed), 1- and 2-way ANOVA with a Holm-Šidak posttest, or log-rank (Mantel-Cox) test for survival curve comparisons. *P* < 0.05 was considered statistically significant.

### Study approval.

Animal experiments were approved by the University of British Columbia Animal Care and Use Committee (A19-0136).

### Data availability.

Values for all data shown in the graphs can be found in the Supporting Data Values file.

## Author contributions

IRS conceived, designed, and conducted the experiments; analyzed the data; and wrote the manuscript. MS conducted the experiments, analyzed the data, and helped critically review the manuscript. MH, KS, and VCWF conducted experiments and critically reviewed the manuscript. DAB provided intellectual input and critically reviewed the manuscript. MM conducted experiments, analyzed the data, provided intellectual input and logistical support, and critically reviewed the manuscript. GR provided intellectual input and critically reviewed the manuscript. MKL secured funding, conceived and designed the experiments, provided overall direction, and wrote the manuscript.

## Supplementary Material

Supplemental data

Supporting data values

## Version 1 09/05/2023

In-Press Preview

## Version 2 10/09/2023

Electronic publication

## References

[1] Lam AJ (2017). Harnessing advances in T regulatory cell biology for cellular therapy in transplantation. Transplantation.

[2] Raffin C (2020). T_reg_ cell-based therapies: challenges and perspectives. Nat Rev Immunol.

[3] Ferreira LMR (2019). Next-generation regulatory T cell therapy. Nat Rev Drug Discov.

[4] Sawitzki B (2020). Regulatory cell therapy in kidney transplantation (the ONE study): a harmonised design and analysis of seven non-randomised, single-arm, phase 1/2A trials. Lancet.

[5] Harden PN (2021). Feasibility, long-term safety, and immune monitoring of regulatory T cell therapy in living donor kidney transplant recipients. Am J Transplant.

[6] Sanchez-Fueyo A (2020). Applicability, safety, and biological activity of regulatory T cell therapy in liver transplantation. Am J Transplant.

[7] Roemhild A (2020). Regulatory T cells for minimising immune suppression in kidney transplantation: phase I/IIa clinical trial. BMJ.

[8] Rosado-Sánchez I, Levings MK (2020). Building a CAR-Treg: going from the basic to the luxury model. Cell Immunol.

[9] Dawson NAJ (2017). Engineered tolerance: tailoring development, function, and antigen-specificity of regulatory T cells. Front Immunol.

[10] Dawson NAJ (2020). Functional effects of chimeric antigen receptor co-receptor signaling domains in human Tregs. Sci Transl Med.

[11] Imura Y (2020). CD19-targeted CAR regulatory T cells suppress B cell pathology without GvHD. JCI Insight.

[12] MacDonald KG (2016). Alloantigen-specific regulatory T cells generated with a chimeric antigen receptor. J Clin Invest.

[13] Boardman DA (2017). Expression of a chimeric antigen receptor specific for donor HLA class I enhances the potency of human regulatory T cells in preventing human skin transplant rejection. Am J Transplant.

[14] Noyan F (2017). Prevention of allograft rejection by use of regulatory T cells with an MHC-specific chimeric antigen receptor. Am J Transplant.

[15] Sicard A (2020). Donor-specific chimeric antigen receptor Tregs limit rejection in naive but not sensitized allograft recipients. Am J Transplant.

[16] Boroughs AC (2019). Chimeric antigen receptor costimulation domains modulate human regulatory T cell function. JCI Insight.

[17] Wagner JC (2022). Anti-HLA-A2-CAR Tregs prolong vascularized mouse heterotopic heart allograft survival. Am J Transplant.

[18] Schreeb K (2022). Study design: human leukocyte antigen class I molecule A^*^02-chimeric antigen receptor regulatory T cells in renal transplantation. Kidney Int Rep.

[19] Guedan S (2018). Engineering and design of chimeric antigen receptors. Mol Ther Methods Clin Dev.

[20] Rafiq S (2020). Engineering strategies to overcome the current roadblocks in CAR T cell therapy. Nat Rev Clin Oncol.

[21] Boardman DA, Levings MK (2022). Emerging strategies for treating autoimmune disorders with genetically modified Treg cells. J Allergy Clin Immunol.

[22] Søndergaard H (2013). Human T cells depend on functional calcineurin, tumour necrosis factor-α and CD80/CD86 for expansion and activation in mice. Clin Exp Immunol.

[23] Walsh NC (2017). Humanized mouse models of clinical disease. Annu Rev Pathol.

[24] Kenney LL (2016). Humanized mouse models for transplant immunology. Am J Transplant.

[25] Shultz LD (2019). Humanized mouse models of immunological diseases and precision medicine. Mamm Genome.

[26] Vignali DAA (2008). How regulatory T cells work. Nat Rev Immunol.

[27] Qureshi OSZ (2011). Trans-endocytosis of CD80 and CD86: a molecular basis for the cell-extrinsic function of CTLA-4. Science.

[28] Tekguc M (2021). Treg-expressed CTLA-4 depletes CD80/CD86 by trogocytosis, releasing free PD-L1 on antigen-presenting cells. Proc Natl Acad Sci U S A.

[29] Liang B (2008). Regulatory T cells inhibit dendritic cells by lymphocyte activation gene-3 engagement of MHC class II. J Immunol.

[30] Akkaya B (2019). Regulatory T cells mediate specific suppression by depleting peptide-MHC class II from dendritic cells. Nat Immunol.

[31] Veldhoen M (2006). Modulation of dendritic cell function by naive and regulatory CD4+ T cells. J Immunol.

[32] Ludwig-Portugall I (2008). Cutting edge: CD25+ regulatory T cells prevent expansion and induce apoptosis of B cells specific for tissue autoantigens. J Immunol.

[33] Zhao DM (2006). Activated CD4+CD25+ T cells selectively kill B lymphocytes. Blood.

[34] Grossman WJ (2004). Human T regulatory cells can use the perforin pathway to cause autologous target cell death. Immunity.

[35] Iikuni N (2009). Cutting edge: regulatory T cells directly suppress B cells in systemic lupus erythematosus. J Immunol.

[36] Weingartner E, Golding A (2017). Direct control of B cells by Tregs: an opportunity for long-term modulation of the humoral response. Cell Immunol.

[37] Wollenberg I (2011). Regulation of the germinal center reaction by Foxp3+ follicular regulatory T cells. J Immunol.

[38] Linterman MA (2011). Foxp3+ follicular regulatory T cells control the germinal center response. Nat Med.

[39] Wing JB (2014). Regulatory T cells control antigen-specific expansion of Tfh cell number and humoral immune responses via the coreceptor CTLA-4. Immunity.

[40] Sage PT (2014). The coinhibitory receptor CTLA-4 controls B cell responses by modulating T follicular helper, T follicular regulatory, and T regulatory cells. Immunity.

[41] Wang CJ (2015). CTLA-4 controls follicular helper T-cell differentiation by regulating the strength of CD28 engagement. Proc Natl Acad Sci U S A.

[42] Alegre ML (2016). Antigen presentation in transplantation. Trends Immunol.

[43] Chakraverty R, Sykes M (2007). The role of antigen-presenting cells in triggering graft-versus-host disease and graft-versus-leukemia. Blood.

[44] Lei YM (2019). Skin-restricted commensal colonization accelerates skin graft rejection. JCI Insight.

[45] Loupy A, Lefaucheur C (2018). Antibody-mediated rejection of solid-organ allografts. N Engl J Med.

[46] Loupy A (2012). The impact of donor-specific anti-HLA antibodies on late kidney allograft failure. Nat Rev Nephrol.

[47] Wardell M Christine MNK (2021). Cross talk between human regulatory T cells and antigen-presenting cells: lessons for clinical applications. Eur J Immunol.

[48] Wing JB (2019). Control of regulatory T cells by co-signal molecules. Adv ExpMed Biol.

[49] Fedorov VD (2013). PD-1- and CTLA-4-based inhibitory chimeric antigen receptors (iCARs) divert off-target immunotherapy responses. Sci Transl Med.

[50] Guedan S (2018). Enhancing CAR T cell persistence through ICOS and 4-1BB costimulation. JCI Insight.

[51] Lamarthee B (2021). Transient mTOR inhibition rescues 4-1BB CAR-Tregs from tonic signal-induced dysfunction. Nat Commun.

[52] Muller YD (2021). The CD28-transmembrane domain mediates chimeric antigen receptor heterodimerization with CD28. Front Immunol.

[53] Hombach AA (2007). Effective proliferation of human regulatory T cells requires a strong costimulatory CD28 signal that cannot be substituted by IL-2. J Immunol.

[54] Tang Q (2003). Cutting edge: CD28 controls peripheral homeostasis of CD4+CD25+ regulatory T cells. J Immunol.

[55] Gogishvili T (2013). Cell-intrinsic and -extrinsic control of Treg-cell homeostasis and function revealed by induced CD28 deletion. Eur J Immunol.

[56] Golovina TN (2008). CD28 costimulation is essential for human T regulatory expansion and function. J Immunol.

[57] Hoeppli RE (2015). The environment of regulatory T cell biology: cytokines, metabolites, and the microbiome. Front Immunol.

[58] Bour-Jordan H, Bluestone JA (2009). Regulating the regulators: costimulatory signals control the homeostasis and function of regulatory T cells. Immunol Rev.

[59] Takeda I (2004). Distinct roles for the OX40-OX40 ligand interaction in regulatory and nonregulatory T cells. J Immunol.

[60] Valzasina B (2005). Triggering of OX40 (CD134) on CD4(+)CD25+ T cells blocks their inhibitory activity: a novel regulatory role for OX40 and its comparison with GITR. Blood.

[61] Vu MD (2007). OX40 costimulation turns off Foxp3+ Tregs. Blood.

[62] Buchan SL (2018). Antibodies to costimulatory receptor 4-1BB enhance anti-tumor immunity via T regulatory cell depletion and promotion of CD8 T cell effector function. Immunity.

[63] Piconese S (2008). OX40 triggering blocks suppression by regulatory T cells and facilitates tumor rejection. J Exp Med.

[64] Choi BK (2004). 4-1BB-dependent inhibition of immunosuppression by activated CD4+CD25+ T cells. J Leukoc Biol.

[65] Hou AJ (2021). Navigating CAR-T cells through the solid-tumour microenvironment. Nat Rev Drug Discov.

[66] Capece D (2012). Targeting costimulatory molecules to improve antitumor immunity. J Biomed Biotechnol.

[67] Zou W, Chen L (2008). Inhibitory B7-family molecules in the tumour microenvironment. Nat Rev Immunol.

[68] Eshhar Z (1993). Specific activation and targeting of cytotoxic lymphocytes through chimeric single chains consisting of antibody-binding domains and the gamma or zeta subunits of the immunoglobulin and T-cell receptors. Proc Natl Acad Sci U S A.

[69] Brocker T, Karjalainen K (1995). Signals through T cell receptor-zeta chain alone are insufficient to prime resting T lymphocytes. J Exp Med.

[70] Brocker T (2000). Chimeric Fv-zeta or Fv-epsilon receptors are not sufficient to induce activation or cytokine production in peripheral T cells. Blood.

[71] Krause A (1998). Antigen-dependent CD28 signaling selectively enhances survival and proliferation in genetically modified activated humanprimary T lymphocytes. J Exp Med.

[72] Sterner RC, Sterner RM (2021). CAR-T cell therapy: current limitations and potential strategies. Blood Cancer J.

[73] Dai H (2022). Treg suppression of immunity within inflamed allogeneic grafts. JCI Insight.

[74] Zhong XS (2010). Chimeric antigen receptors combining 4-1BB and CD28 signaling domains augment PI 3 kinase/AKT/Bcl-X L activation and CD8 T cell-mediated tumor eradication. Mol Ther.

[75] Friedmann-Morvinski D (2005). Redirected primary T cells harboring a chimeric receptor require costimulation for their antigen-specific activation. Blood.

[76] Kashem SW (2017). Antigen-presenting cells in the skin. Annu Rev Immunol.

[77] Scheib N (2022). The dendritic cell dilemma in the skin: between tolerance and immunity. Front Immunol.

[78] Benichou G (2011). Immune recognition and rejection of allogeneic skin grafts. Immunotherapy.

[79] Debes GF, McGettigan SE (2019). Skin-associated B cells in health and inflammation. J Immunol.

[80] Zhang N (2009). Regulatory T cells sequentially migrate from inflamed tissues to draining lymph nodes to suppress the alloimmune response. Immunity.

[81] Morelli AE (2014). Dendritic cells of myeloid lineage: the masterminds behind acute allograft rejection. Curr Opin Organ Transplant.

[82] Liu Q (2016). Donor dendritic cell-derived exosomes promote allograft-targeting immune response. J Clin Invest.

[83] Prunevieille A (2021). T cell antigenicity and immunogenicity of allogeneic exosomes. Am J Transplant.

[84] Zeng F (2021). Graft-derived extracellular vesicles transported across subcapsular sinus macrophages elicit B cell alloimmunity after transplantation. Sci Transl Med.

[85] Lee K (2014). Attenuation of donor-reactive T cells allows effective control of allograft rejection using regulatory T cell therapy. Am J Transplant.

[86] Tsang JY (2008). Conferring indirect allospecificity on CD4+CD25+ Tregs by TCR gene transfer favors transplantation tolerance in mice. J Clin Invest.

[87] Ratnasothy K (2019). IL-2 therapy preferentially expands adoptively transferred donor-specific Tregs improving skin allograft survival. Am J Transplant.

[88] Wu D (2021). A method for expansion and retroviral transduction of mouse regulatory T cells. J Immunol Methods.

[89] Cossarizza A (2021). Guidelines for the use of flow cytometry and cell sorting in immunological studies (third edition). Eur J Immunol.

