# Supplementary material for: Tregs integrate native and CAR-mediated costimulatory signals for control of allograft rejection
Source: JCI Insight. 2023 Oct 9;8(19):e167215. doi: 10.1172/jci.insight.167215 (PMC10619441; doi:10.1172/jci.insight.167215)
Supplement: Supplemental data [file jciinsight-8-167215-s149.pdf]

SUPPLEMENTARY DATA

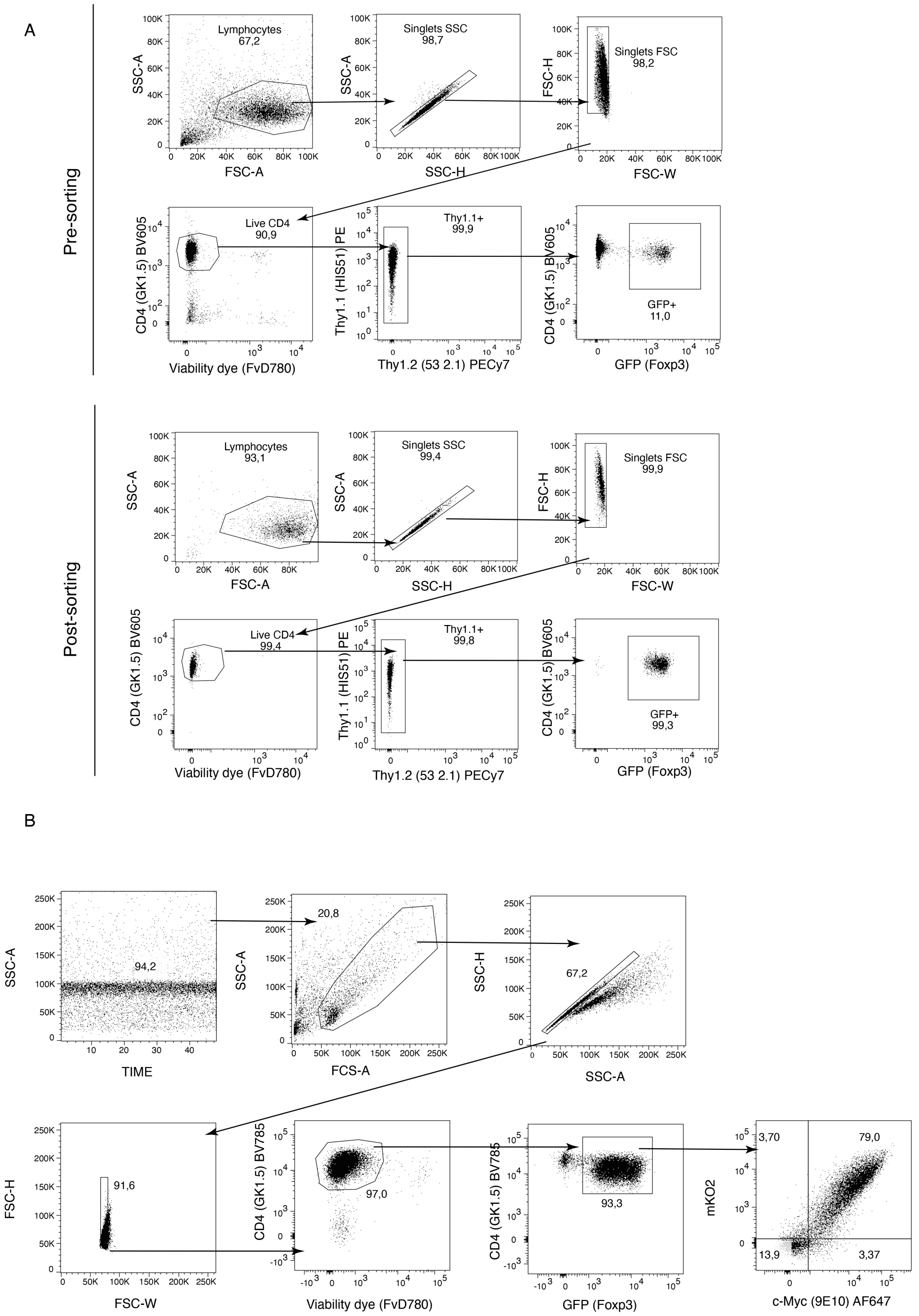

Supplementary Figure 1. *Sorting and purity check flow panels.* (**A**) Representative data of at least 5 independent experiments showing the pre- and post-sorting flow panel used to isolate Tregs (defined as live CD4+CD8-Thy1.1+Foxp3^{gfp}+ cells). (**B**) Representative data of at least 5 independent experiments showing the flow panel used to check Treg purity and CAR-expression after Treg expansion at day 7.

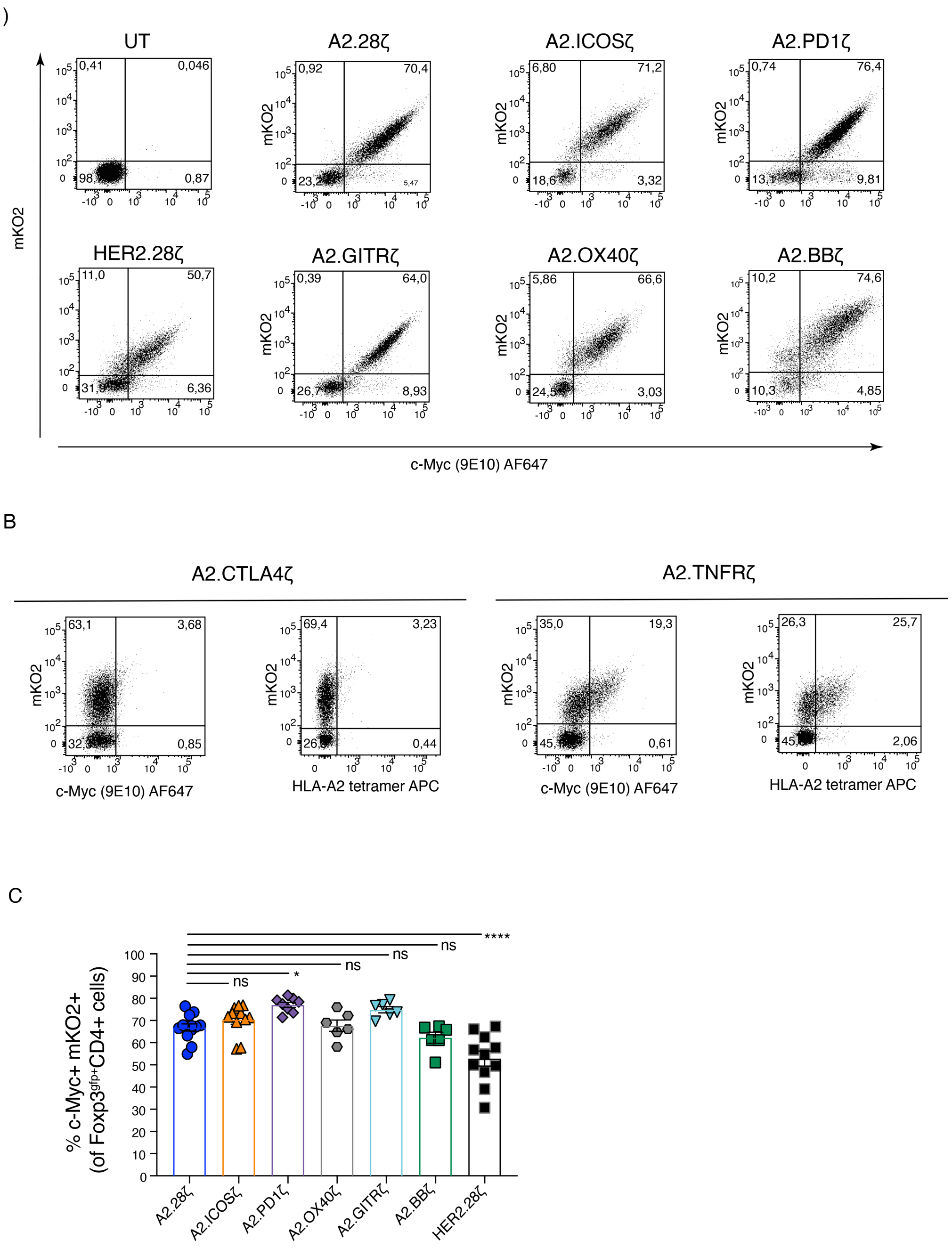

Supplementary Figure 2: *Co-stimulatory domain variant CAR expression:* (**A**) Representative data of at least 5 independent experiments showing the expression of co-stimulatory domain CAR variants defined by c-Myc and mKO2 co-expression in Tregs. (**B**) Lack of CAR expression or binding to HLA-A2 for the CTLA4- and TNFR2-based CAR variants. (**C**) Frequencies of c-Myc and mKO2 co-expression in Tregs, gated in live CD4+foxp3^{gfp}+ cells; n=6 to 13 replicates from at least 8 independent experiments. Data shown as mean±SEM. Statistical significance was determined using one-way ANOVA with a Holm-Sidak post-test, *p<0.05, **p<0.01, ***p<0.001, ****p<0.0001.

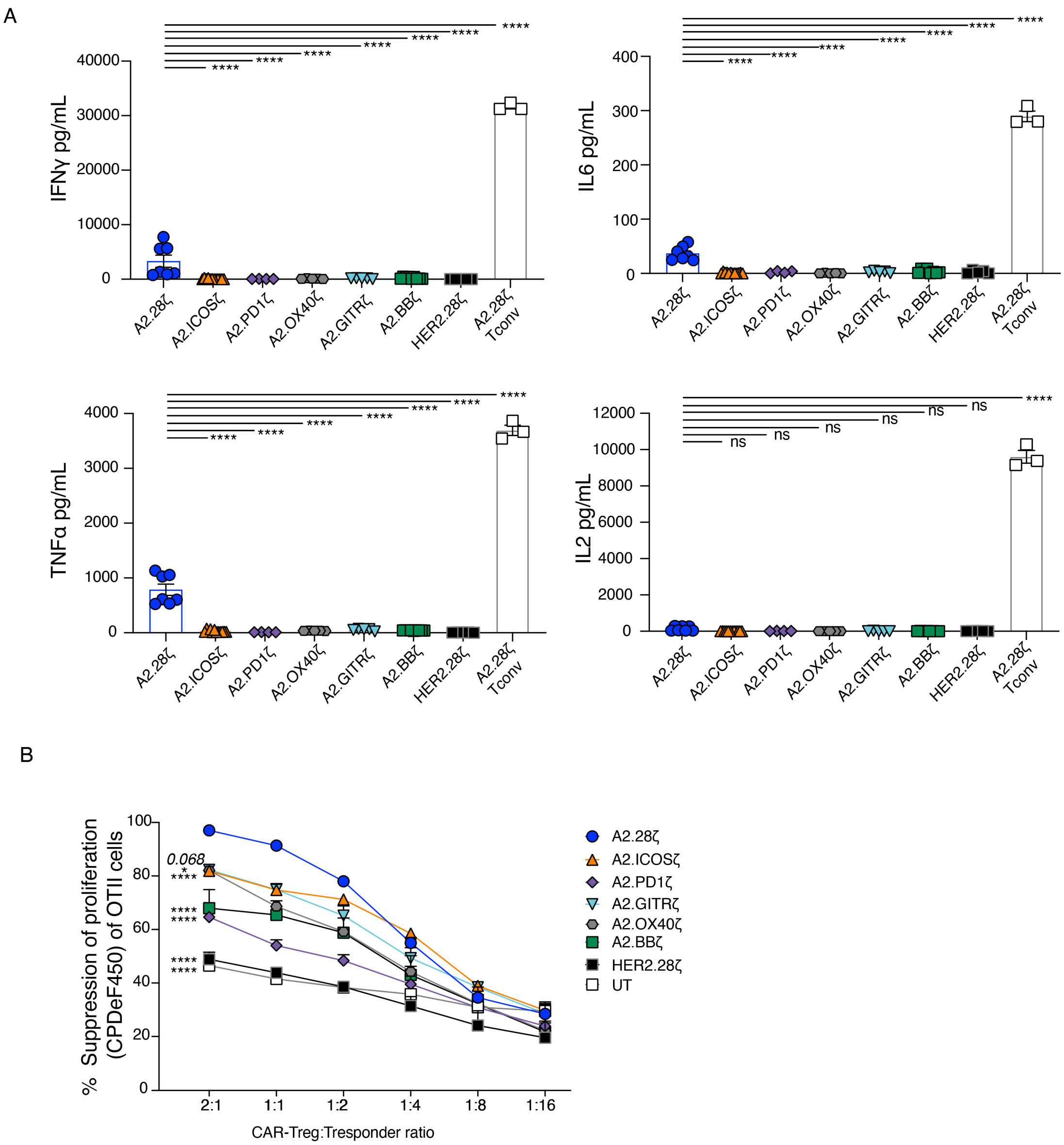

Supplementary Figure 3: *Supplementary data for the in vitro characterization of co-stimulatory domain CAR variants.* (**A**) Cytokine production of CAR-Tregs cultured for 3-days with HLA-A2$^+$ K562 cells; n=3 to 12 replicates from at least 3 independent experiments. (**B**) CAR-Treg suppression of the OTII CD4 T responder proliferation, as determined by CPDeF450 dilution; n=3 to 6 replicates from at least 2 independent experiments. UT = Untransduced. Data shown as mean±SEM. Statistical significance was determined using one-way (**A**) two-way (**B**) ANOVA with a Holm-Sidak post-test. *p<0.05, **p<0.01, ***p<0.001. ****p<0.0001.

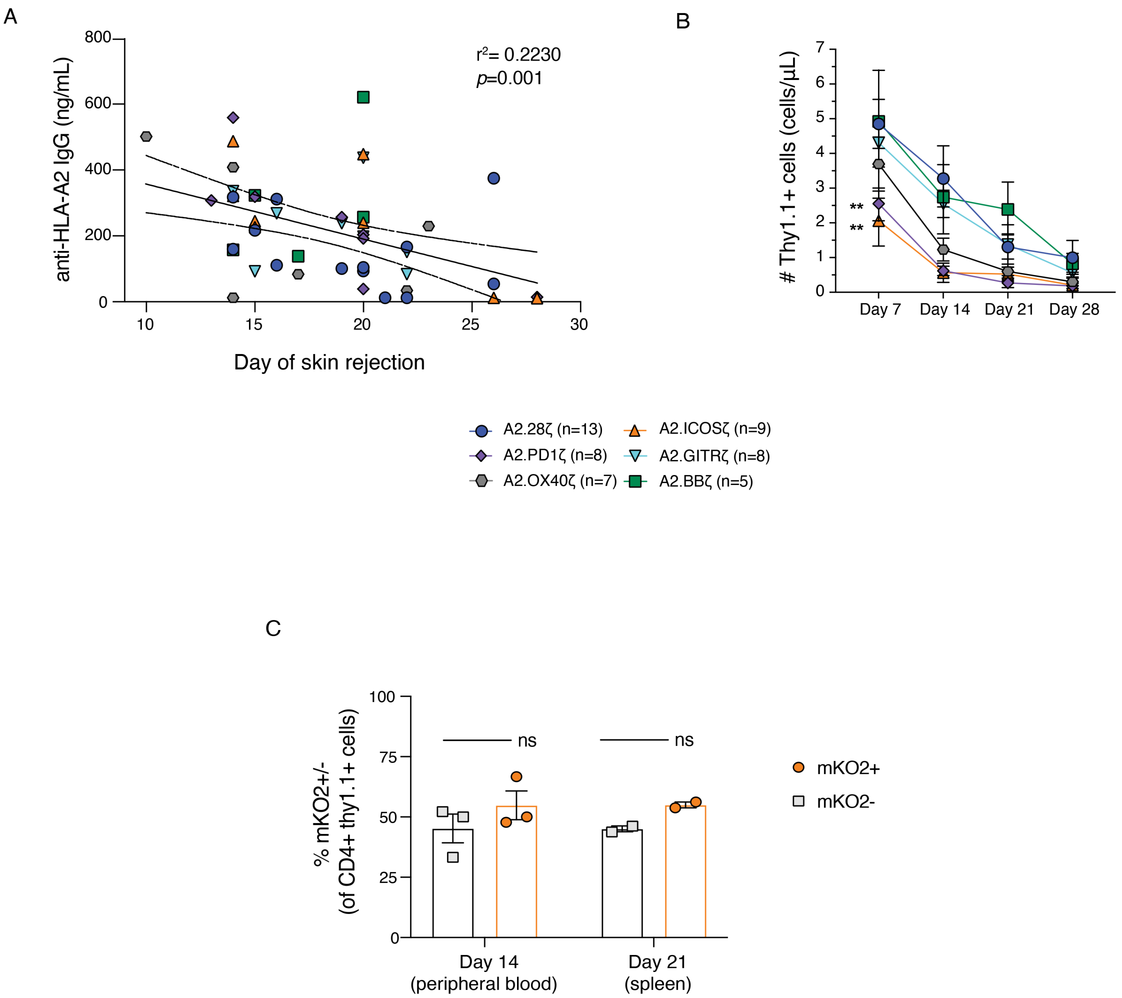

Supplementary Figure 4: *Supplementary data for the in vivo characterization of co-stimulatory domain CAR variants.* (**A**) Correlation between levels of anti-HLA-A2 IgG Abs in plasma at day 14 and skin graft rejection date of mice receiving Tregs bearing CD28 and TNFR family-based co-stimulatory domain CARs. (**B**) Count of CAR-Tregs in peripheral blood of mice transplanted with HLA-A2-expressing skin and intravenously administered with 1x10^6 CAR-Tregs. (**A-B**) n=3 to 13 mice per group from at least 4 individual experiments. (**C**) Relative frequencies of mKO2+ and mKO2- CAR-Tregs in peripheral blood and spleen; n=3 mice per group from one experiment. BL/6 mice were transplanted with skin grafts from HLA-A2$^+$ BL/6 mice and intravenously administered 5x10^5 mKO2^{pos} and 5x10^5 mKO2^{neg} CD28-bearing CAR-Tregs. Data shown as mean±SEM. Statistical significance was determined using Pearson Correlation (**A**), or two-way ANOVA (**B-C**) with a Holm-Sidak post-test. *p<0.05, **p<0.01, ***p<0.001. ****p<0.0001.

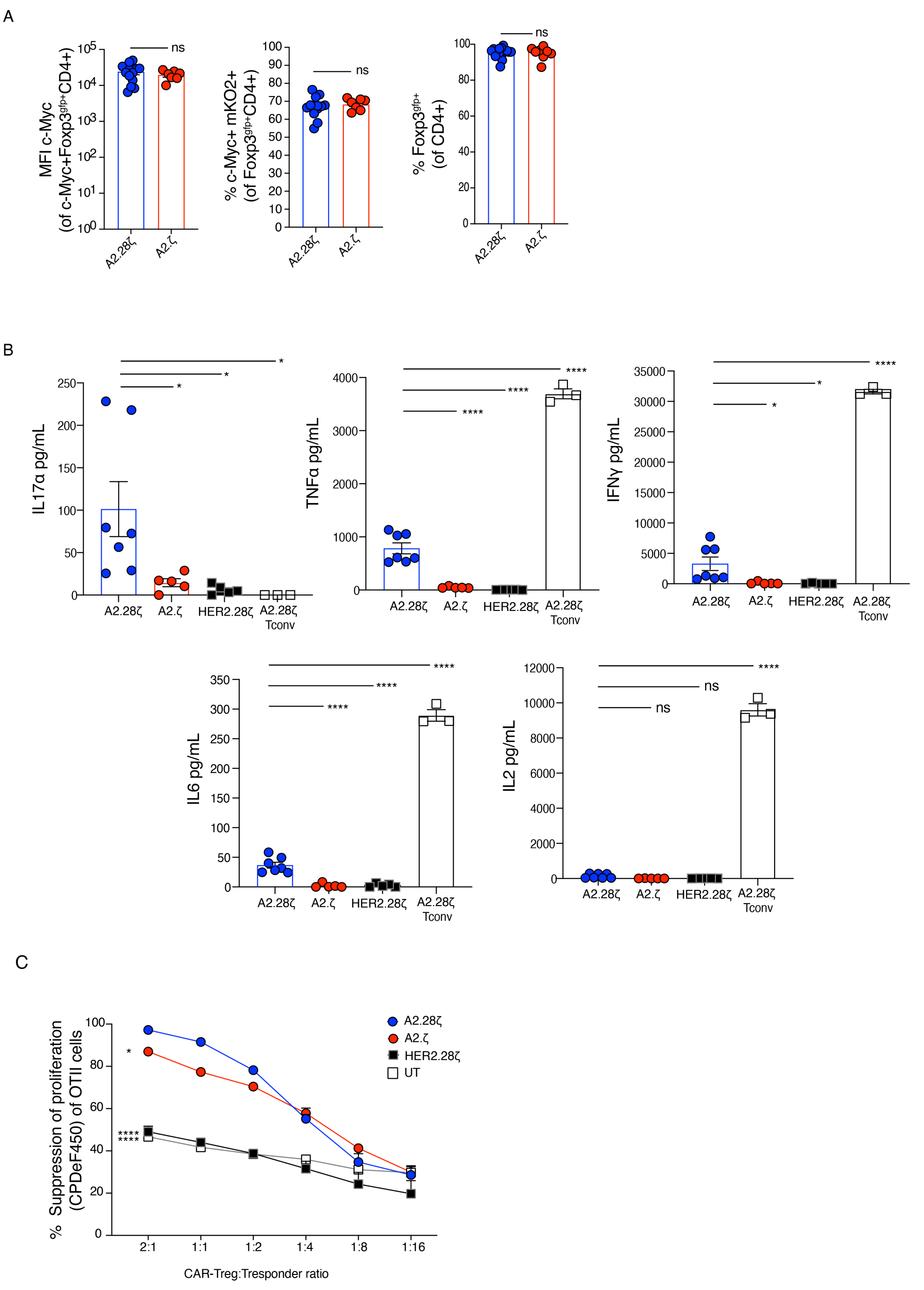

Supplementary Figure 5: *Supplementary material for the in vitro comparison of first- and second-generation CARs*: **A**) MFI of CAR expression, co-expression of Myc and mKO2 and Foxp3^{gfp} expression for first- and second-generation CAR-Tregs after expansion, gated on total live Myc+CD4^{+}Foxp3^{gfp+} or live CD4^{+} cells; n=7 to 16 replicates from at least 8 independent experiments. (**B**) Cytokine production of CAR-Tregs cultured for 3-days with HLA-A2^{pos} K562 cells; n=3 to 7 replicates from 3 independent experiments (**C**) CAR-Treg suppression of the OTII CD4 T responder proliferation, as determined by CPDeF450 dilution; n=3 to 6 replicates from 2 independent experiments UT = Untransduced. Data shown as mean±SEM. Statistical significance was determined using t-student (**A**), one-way (**B**) two-way ANOVA (**C**) with a Holm-Sidak post-test. *p<0.05, **p<0.01, ***p<0.001. ****p<0.0001.

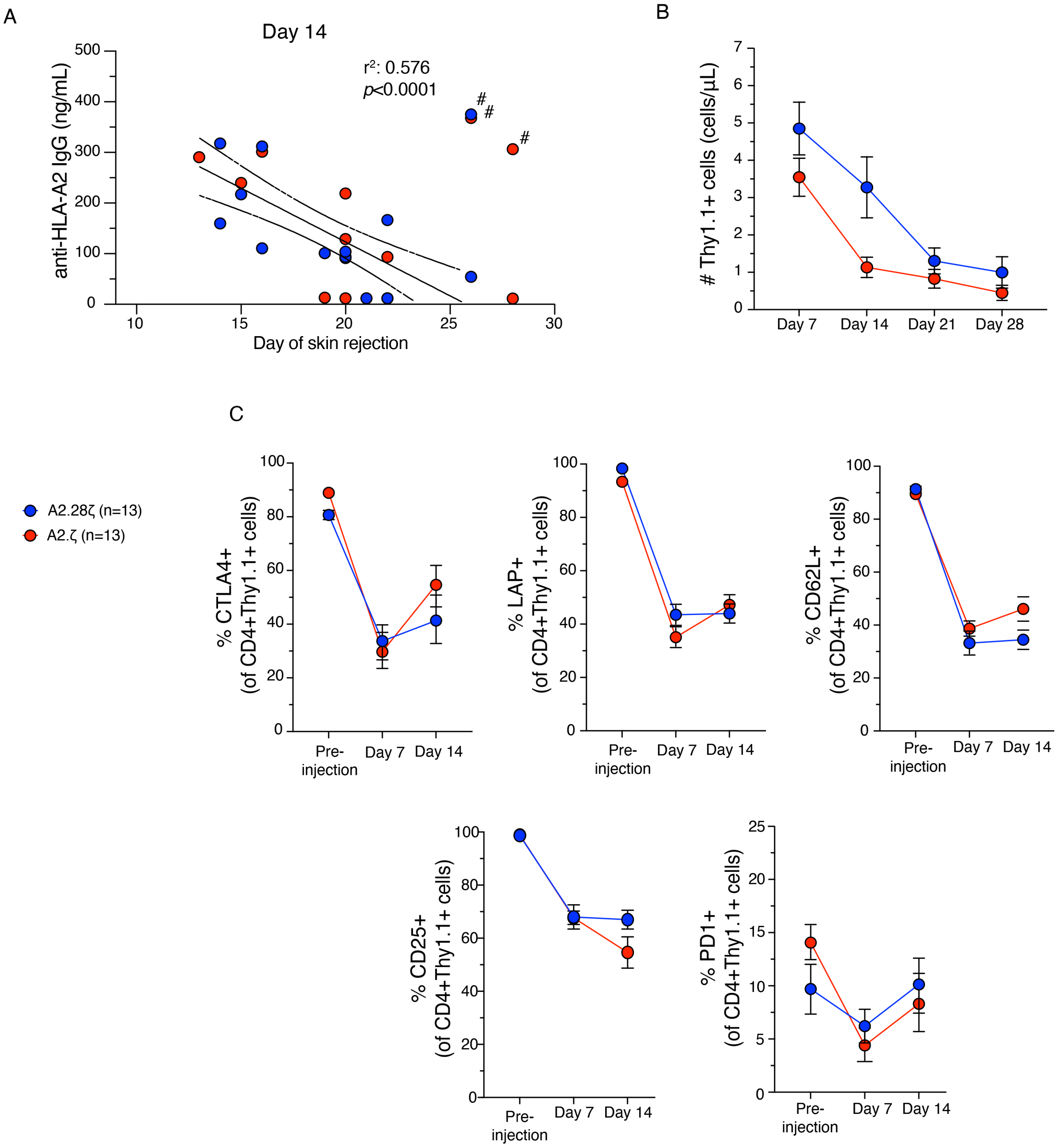

Supplementary Figure 6: *Supplementary material for the in vivo comparison of first- and second-generation CARs*. BL/6 mice were transplanted with skin grafts from syngeneic or HLA-A2$^+$ BL/6 mice and were intravenously administered 1x10^6 CAR-Tregs. (**A**) Correlation between anti-HLA-A2 IgG Abs in plasma at day 14 and skin graft rejection from mice receiving Tregs bearing A2.28ζ or A2.ζ CARs. # Outliers values excluded from linear regression analysis. (**B**) Counts of Thy1.1$^+$ CAR-Tregs of total CD45$^+$ T cells in peripheral blood over time. (**C**) Phenotype of Thy1.1$^+$CD4$^+$ CAR-Tregs in peripheral blood over time including expression of CTLA-4, LAP, CD62L, CD25 and PD1. Data pooled from 3 individual in vivo experiments with n=3 to 13 mice per group and shown as mean±SEM. Statistical significance was determined using Pearson Correlation (**A**) and two-way ANOVA with a Holm-Sidak post-test (**B,C**). *p<0.05, **p<0.01, ***p<0.001. ****p<0.0001.

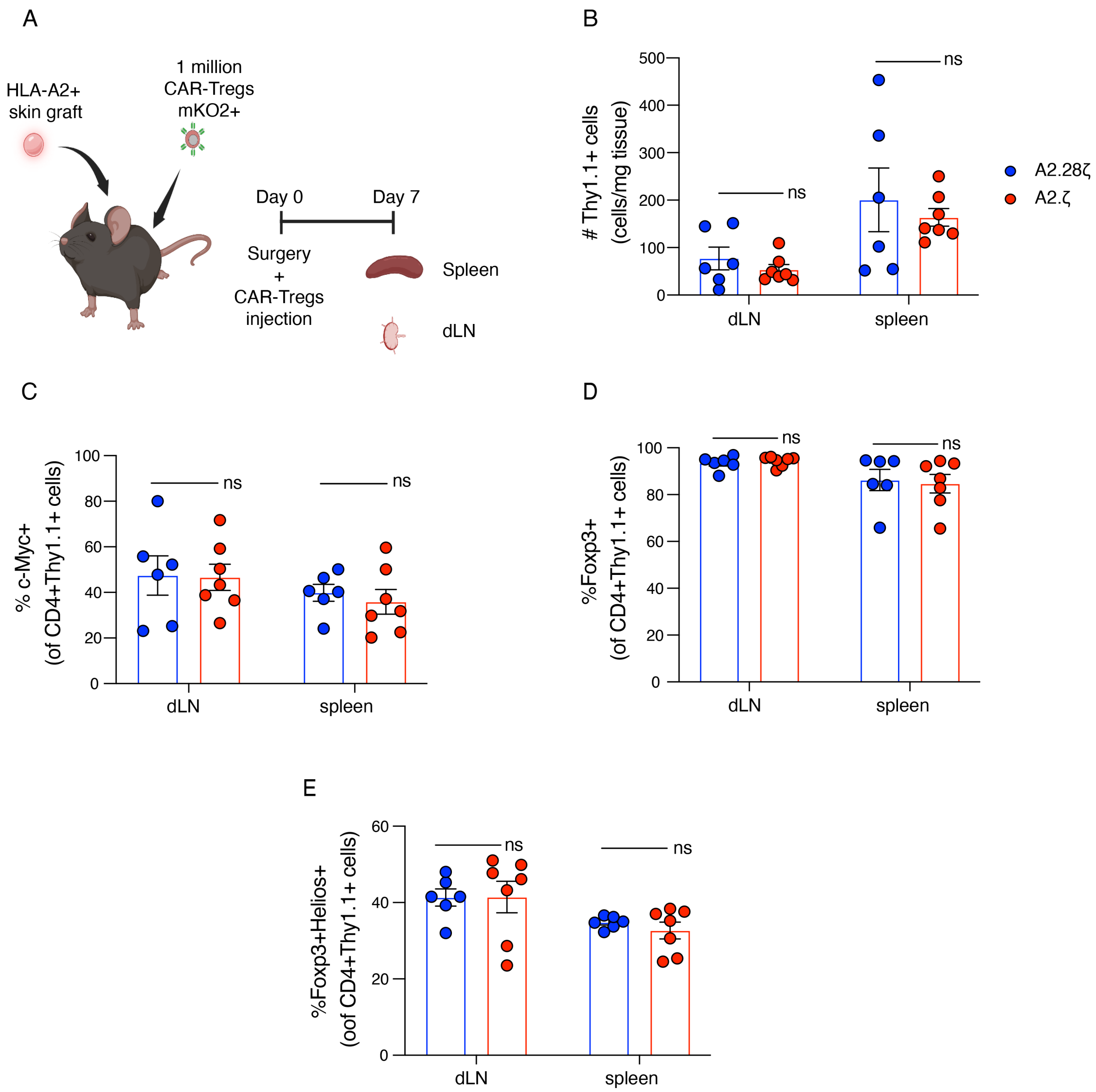

Supplementary Figure 7: *Study of first- and second-generation CAR-Tregs in lymphoid tissues.* (**A**) Schematic diagram of the *study of first- and second-generation CAR-Tregs in lymphoid tissues.* BL/6 mice were transplanted with skin grafts from HLA-A2^{+} BL/6 mice and intravenously administered 1x10^{6} CAR-Tregs. Spleen and draining lymphoid node (dLN) were collected at day 7 post-infusion. (**B**) Numbers of *first- and second-generation CAR-Tregs in dLN (left) and spleen (right). **(C-F)*** CAR-Tregs expression of: (**C**) CAR (c-Myc+) (**D**), FoxP3 and (**E**) FoxP3 and Helios in *dLN and spleen.* Data are mean±SEM generated from two independent experiments; n=6 to 7 mice per group. Statistical significance was determined using Student's t-test with a Holm-Sidak post-test, *p<0.05, **p<0.01, ***p<0.001, ****p<0.0001.

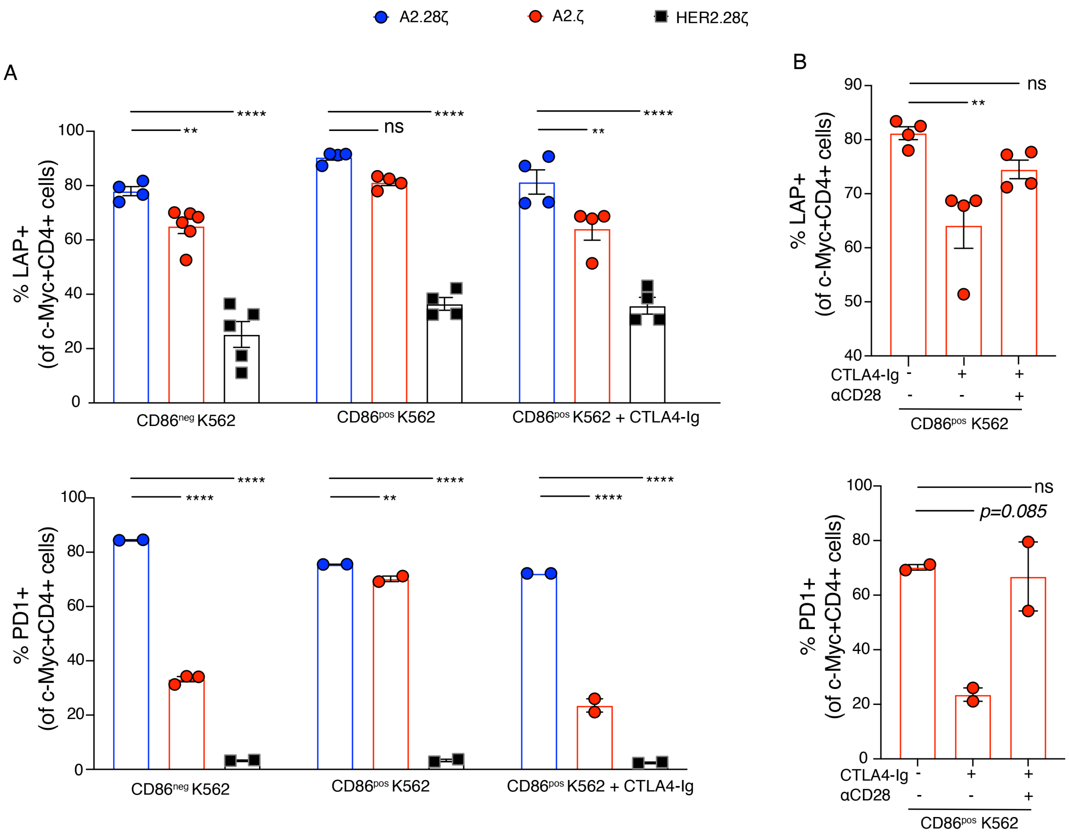

Supplementary Figure 8: *Supplementary material of the effects of a CAR-independent stimulation over the CAR-mediated activation and function of Tregs.* CAR-Tregs were co-cultured with $CD86^{+}HLA\text{-}A2^{+}$ or $CD86^{-}HLA\text{-}A2^{+}$ K562 cells at a 1:2 K562:Tregs ratio for 3 days. (**A-B**) LAP and PD1 expression in CAR-Tregs following 3-days of co-culture, gated on Myc+CD4+ live cells. Where indicated, CTLA4-Ig or activating anti-CD28 mAbs were added at 10 mg/mL. N= 2 to 6 replicates from 2 or 1 independent experiments for LAP and PD1, respectively. Data is shown as mean±SEM. Statistical significance was determined using one-way (**B**) or two-way (**A**) ANOVA with a Holm-Sidak post-test, *p<0.05, **p<0.01, ***p<0.001, ****p<0.0001.

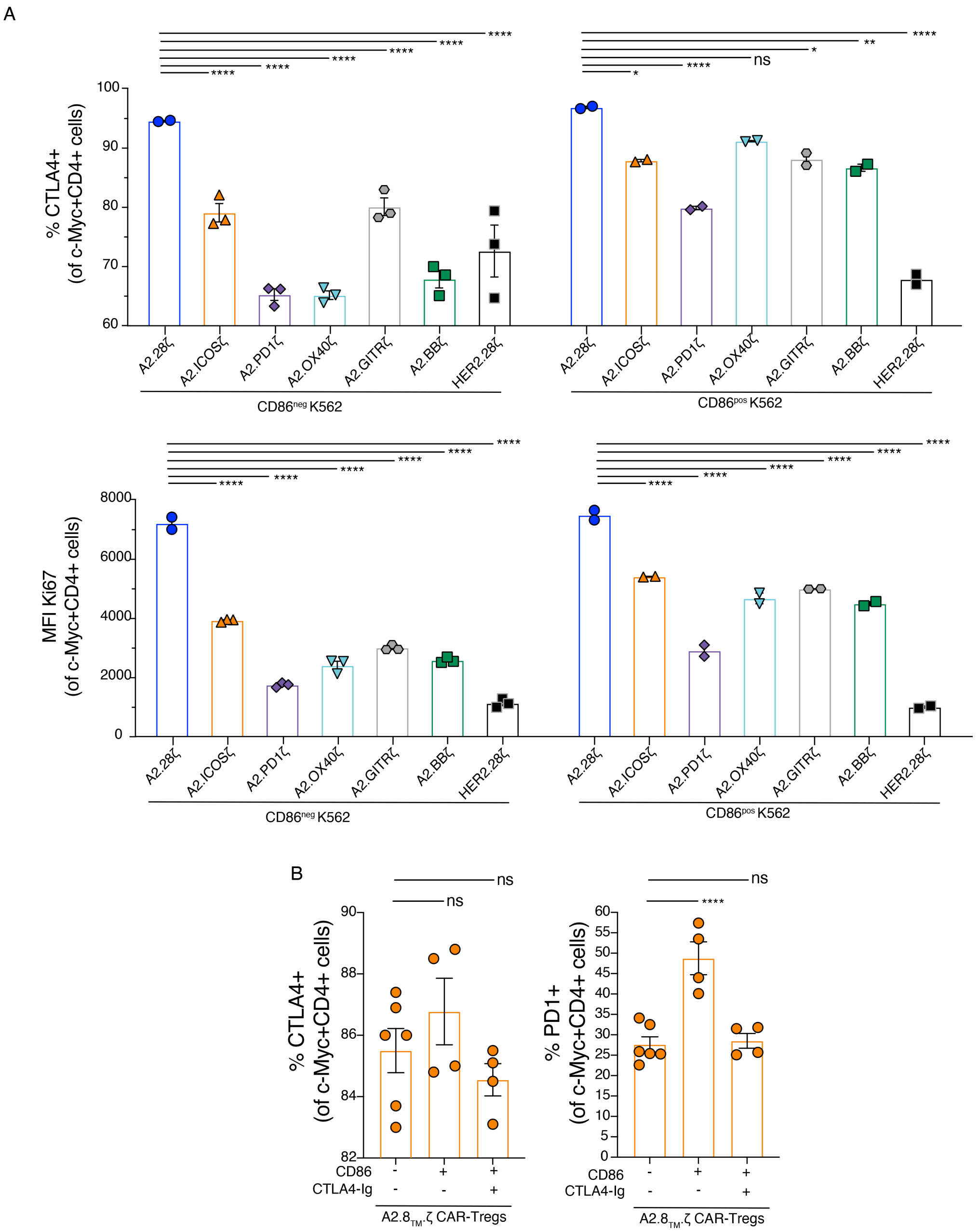

Supplementary Figure 9: *Supplementary material of the effects of a CAR-independent stimulation over the CAR-mediated activation and function of Tregs.* CAR-Tregs were co-cultured with CD86^{+}HLA-A2^{+} or CD86^{-}HLA-A2^{+} K562 cells at a 1:2 K562:Tregs ratio for 3 days. (**A**) CTLA4 and Ki67 expression in different co-stimulatory CAR-Tregs following 3-days of co-culture, gated on Myc+CD4+ live cells; n=2 to 3 replicates from one experiment. (**B**) CTLA4 and PD1 expression in CD8α-TM CAR-Tregs following 3-days of co-culture, gated on Myc+CD4+ live cells; n=4 to 6 replicates from one experiment. Where indicated, CTLA4-Ig or activating anti-CD28 mAbs were added at 10 μg/mL. Data are shown as mean±SEM. Statistical significance was determined using one-way (**B**) or two-way (**A**) ANOVA with a Holm-Sidak post-test, *p<0.05, **p<0.01, ***p<0.001, ****p<0.0001.

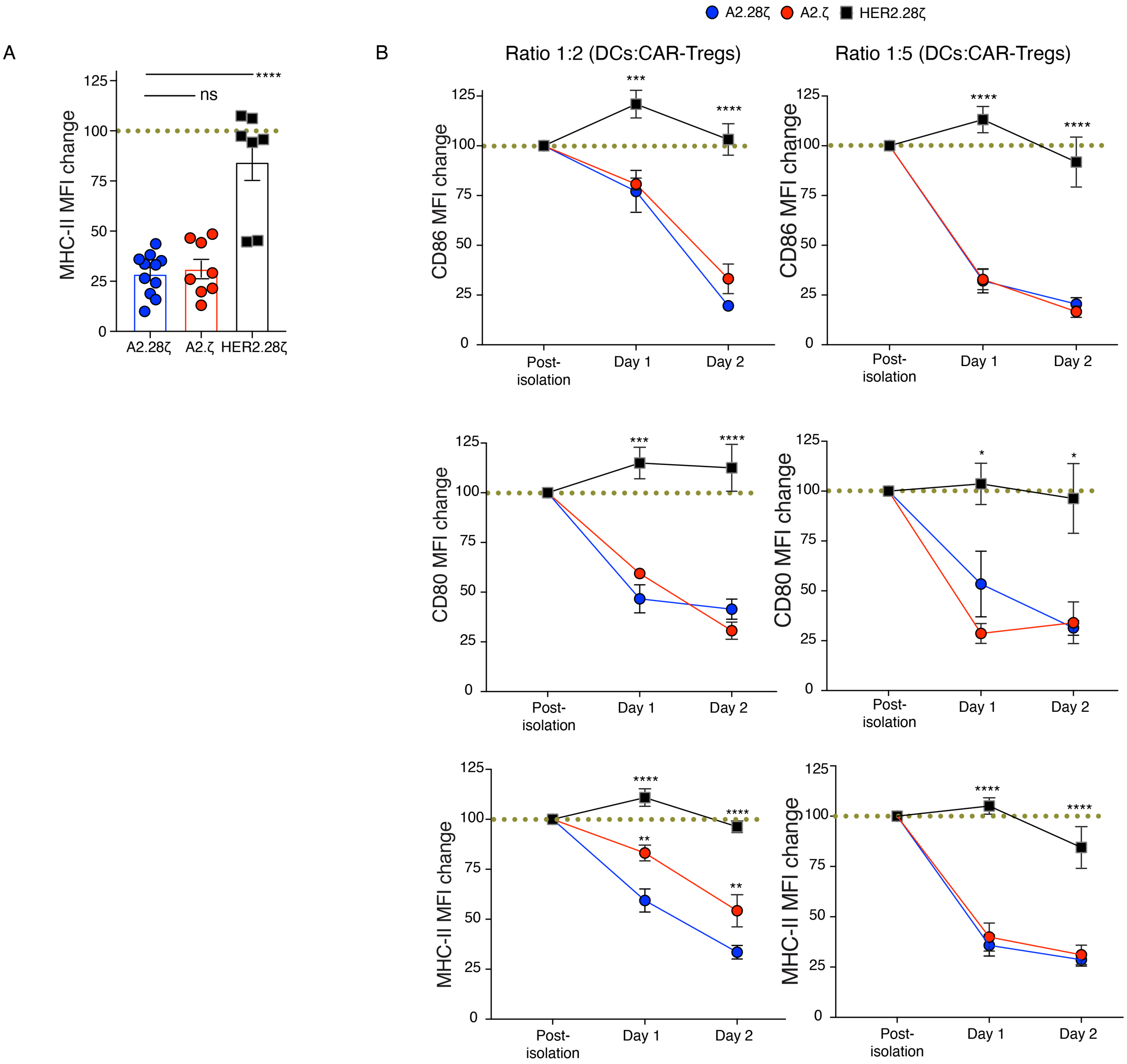

Supplementary Figure 10: *Supplementary material of the in vitro APC suppression by first- and second-generation CAR-Tregs.* CAR-Tregs were co-cultured with splenic HLA-A2^{+} CD11c^{+} DCs at a 1:2 or 1:5 DCs:Tregs for 1 or 2 days. (**A**) Expression of MHC-II in HLA-A2+ CD11c^{+} DCs after 2 days of culture with the indicated CAR-Tregs; n=7 to 11 replicates from at least 4 independent experiments. (**B**) Expression of CD80, CD86 and MHC-II in HLA-A2+ CD11c^{+} DCs cultured at different time points and with different ratios of DCs/CAR-Tregs; n=6 to 15 replicates from at least 4 independent experiments. Data are shown relative to DCs cultured with untransduced Tregs which were normalized to 100% (dotted lines). Data are shown as mean±SEM. Statistical significance was determined using one-way (**A**) or two-way (**B**) ANOVA with a Holm-Sidak post-test, *p<0.05, **p<0.01, ***p<0.001, ****p<0.0001.

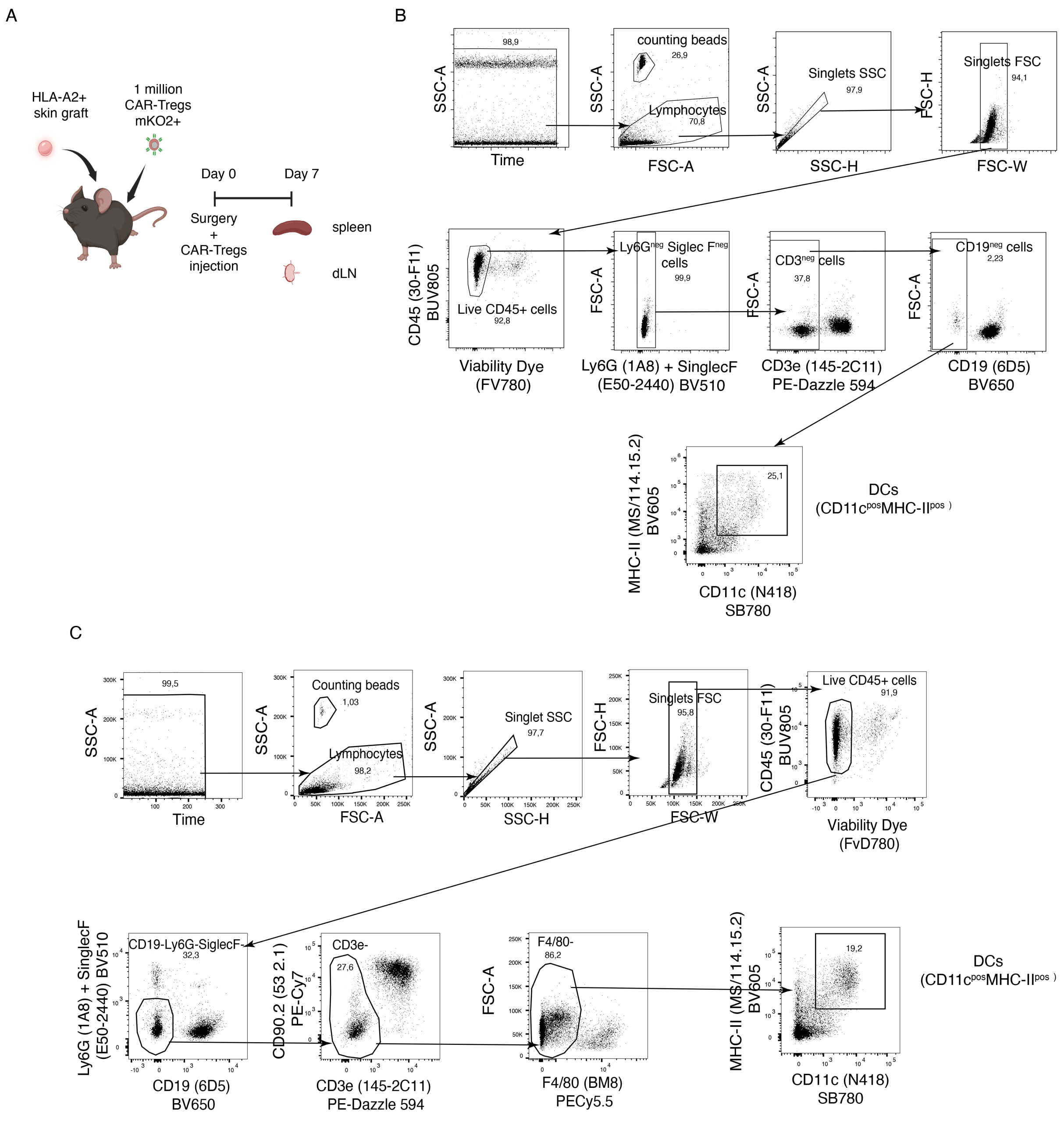

Supplementary Figure 11: *Study design and flow panels of the in vivo study of CAR-Treg modulation of co-stimulatory and MHC-II molecules expression in APCs.* (**A**) Schematic diagram of the study of first- and second-generation CAR-Tregs modulation of *co-stimulatory and MHC-II molecules expression in APCs*. BL/6 mice were transplanted with skin grafts from HLA-A2^{+} BL/6 mice and intravenously administered 1x10^{6} CAR-Tregs. Spleen and draining lymphoid node (dLN) were collected at day 7 post-infusion. (**B**) Representative data of one experiment showing the flow panel used to study dendritic cells (DCs) in dLNs (defined as live CD45^{pos}Ly6G^{neg}SigletF^{neg}CD3e^{neg}CD19^{neg}CD11c^{pos}MHCII^{pos}). (**C**) Representative data of one experiment showing the flow panel used to study DCs in spleen (defined as live CD45^{pos}Ly6G^{neg}SigletF^{neg}CD19^{neg}CD3e^{neg}F4/80^{neg}CD11c^{pos}MHCII^{pos}).

Supplementary Table 1

CAR	Specificity	Hinge	Transmembrane domain	Costimulatory domain
A2.28ζ	HLA-A2	CD8α-derived	FWALVVVAGVLFCYGLLVTVALCVIWT	NSRRNRLLQSDYMNMTPRRPGLTRKPYQPYAPARDFAAYRP
A2.ICOSζ	HLA-A2	CD8α-derived	PVGCAAFVVVLLFGCILIIWF	SKKKYGSSVHDPNSEYMFMAAVNTNKKSRLAGVTS
A2.PD1ζ	HLA-A2	CD8α-derived	FWALVVVAGVLFCYGLLVTVALCVIWT	AVFCSTSMSEARGAGSKDDTLKEEPSAAPVPSVAYEELDFQGREKTPELPTACVHTEYATIVFTEGLGASAMGRRGSADGLQGPRPPRHEDGHCSWPL
A2.CTLA4ζ	HLA-A2	CD8α-derived	FWALVVVAGVLFCYGLLVTVALCVIWT	AVSLSKMLKKRSPLTTGVYVKMPPTEPECEKQFQPYFIPIN
A2.GITRζ	HLA-A2	CD8α-derived	LTVIFLVMAACIFFLTTVQLG	LHIWQLRRQHMCPRETQPFAEVQLSAEDACSFQFPEEERGEQTEEKCHLGGRWP
A2.OX40ζ	HLA-A2	CD8α-derived	AFAVLLGLGLGLLAPLTVLLALYLL	RKAWRLPNTPKPCWGNSFRTPIQEEHTDAHFTLAKI
A2.BBζ	HLA-A2	CD8α-derived	TLFLALTSALLLALIFITLLF	SVLKWIRKKFPHIFKQPFKKTTGAAQEEDACSCRCPQEEEGGGGGYEL
A2.TNFRζ	HLA-A2	CD8α-derived	ISLPIGLIVGVTSLGLLMLGLVNCIILVQR	KKKPSCLQRDAKVPHVPDEKSQDAVGLEQQHLLTTAPSSSSSSLESSASAGDRRAPPGGHPQARVMAEAQGFQEARASSRISDSSHGSHGTHVNVTCIVNVCSSSDHSSQCSSQASATVGDPDAKPSASPKDEQVPFSQEECPSQSPCETTETLQSHEKPLPLGVPDMGMKPSQAGWFDQIAVKVA
A2.ζ	HLA-A2	CD8α-derived	FWALVVVAGVLFCYGLLVTVALCVIWT	
HER2.28ζ	HER2	CD8α-derived	FWALVVVAGVLFCYGLLVTVALCVIWT	NSRRNRLLQSDYMNMTPRRPGLTRKPYQPYAPARDFAAYRP
A2.8$_{TM}$.28ζ	HLA-A2	CD8α-derived	IWAPLAGICVALLLSLIITLI	NSRRNRLLQSDYMNMTPRRPGLTRKPYQPYAPARDFAAYRP
A2.8$_{TM}$. ζ	HLA-A2	CD8α-derived	IWAPLAGICVALLLSLIITLI	
HER2.8$_{TM}$.28ζ	HER2	CD8α-derived	IWAPLAGICVALLLSLIITLI	NSRRNRLLQSDYMNMTPRRPGLTRKPYQPYAPARDFAAYRP

Supplementary Table 2: List of antibodies and other reagents used in the study

Target	Clone	Fluorophore	Company
CD4	GK1.5	BV785	BD Biosciences
CD4	RM4-5	BUV563	BD Biosciences
CD45	30-F11	BUV805	BD Biosciences
LAP	Tw7-16B4	PerCP-e710	BD Biosciences
PD1	RMP1-30	BV711	BD Biosciences
CD25	PC61	BUV395	BD Biosciences
CD80	16-10A1	FITC or BUV395	BD Biosciences
CD86	GL1	APC	BD Biosciences
CD8	53-6.7	PE or AF647	BD Biosciences
CD3e	145-2C11	PE-Dazzle 594	BD Biosciences
Ly6G	1A8	BV510	BD Biosciences
Siglec F	E50-2440	BV510	BD Biosciences
Thy1.1	HIS51	PE or BUV496	eBioscience
Thy1.2	53 2.1	BV510 or PECy7	eBioscience
Ki67	SolA15	AF700	eBioscience
FoxP3	FJK-16s	FITC or PECy7	eBioscience
CD11c	N418	SB780	eBioscience
Helios	22F6	PE-Dazzle 594 or eF450	Biolegend
F4/80	BM8	PE-Cy5	Biolegend
CTLA4	UC10-4B9	BV605	Biolegend
I-A/I-E (MHC-II)	MS/114.15.2	BV605	Biolegend
CD62L	MEL-14	AF647	Biolegend
CD19	6D5	BV650	Biolegend
cMyc	9E10	AF647 or AF488	UBC Ablab
Streptavidin		APC	BD Biosciences
Streptavidin		PE	BD Biosciences
HLA-A2:01 Biotin monomer			NIH Tetramer Core Facility
Goat anti-Mouse IgG (H+L)	Polyclonal	APC	Invitrogen
